# Supplementary material for: Four-dimensional trapped ion mobility spectrometry lipidomics for high throughput clinical profiling of human blood samples
Source: Nat Commun. 2023 Feb 20;14:937. doi: 10.1038/s41467-023-36520-1 (PMC9941096; doi:10.1038/s41467-023-36520-1)
Supplement: Supplementary file 1 — Supplementary Information [file 41467_2023_36520_MOESM1_ESM.pdf]

SUPPLEMENTARY INFORMATION

Four-dimensional trapped ion mobility spectrometry lipidomics for high throughput clinical profiling of human blood samples

Raissa Lerner[1#], Dhanwin Baker[1#], Claudia Schwitter[1], Sarah Neuhaus[1], Tony Hauptmann[2], Julia M. Post[1], Stefan Kramer[2], Laura Bindila[1*]

[1]Clinical Lipidomics Unit, Institute of Physiological Chemistry, University Medical Center, Duesbergweg 6, 55128 Mainz, Germany

[2]Data mining, Institute of computer science, Johannes Gutenberg University Mainz, Staudingerweg 9, 55128 Mainz, Germany

Supplementary Figure 1

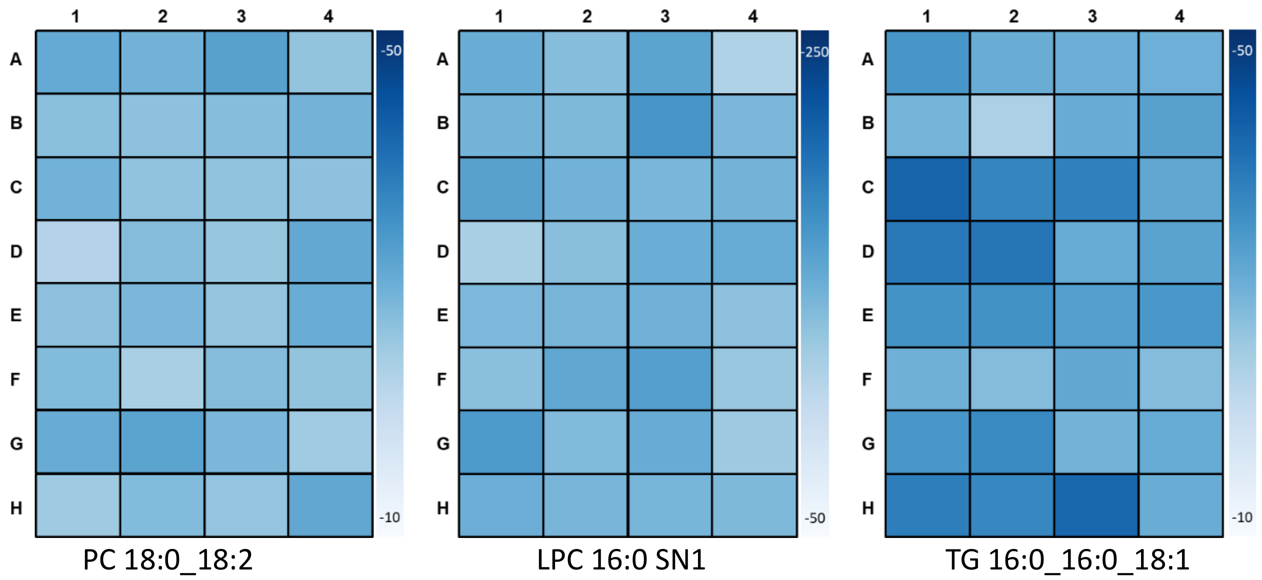

Supplementary Figure 1. Distribution of lipid features and intensities. Comparison of log10 of quantified values (nmol/mL) for phosphatidylcholine (PC) PC 18:0_18:2, lysophosphatidylcholine (LPC) LPC 16:0 SN1, and triglyceride (TG) TG 16:0_16:0_18:1 in 32 samples injected from spot A1 to spot H4. The color bar of each subpanel corresponds to a concentration range, with light blue representing lower and dark blue representing higher calculated concentrations. Source data are provided as a Source Data file.

Supplementary Figure 2

a

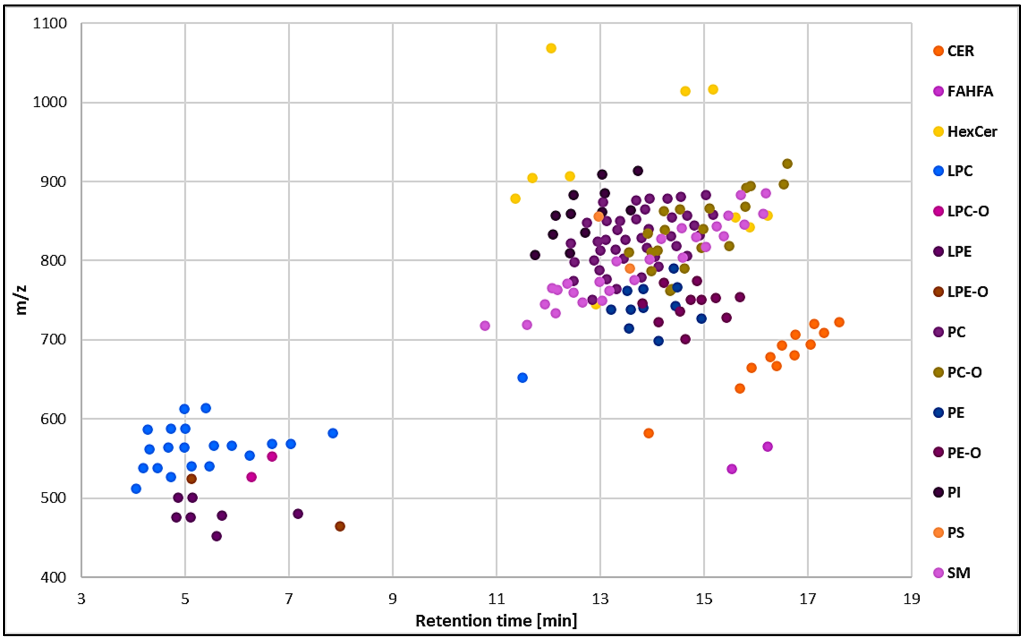

b

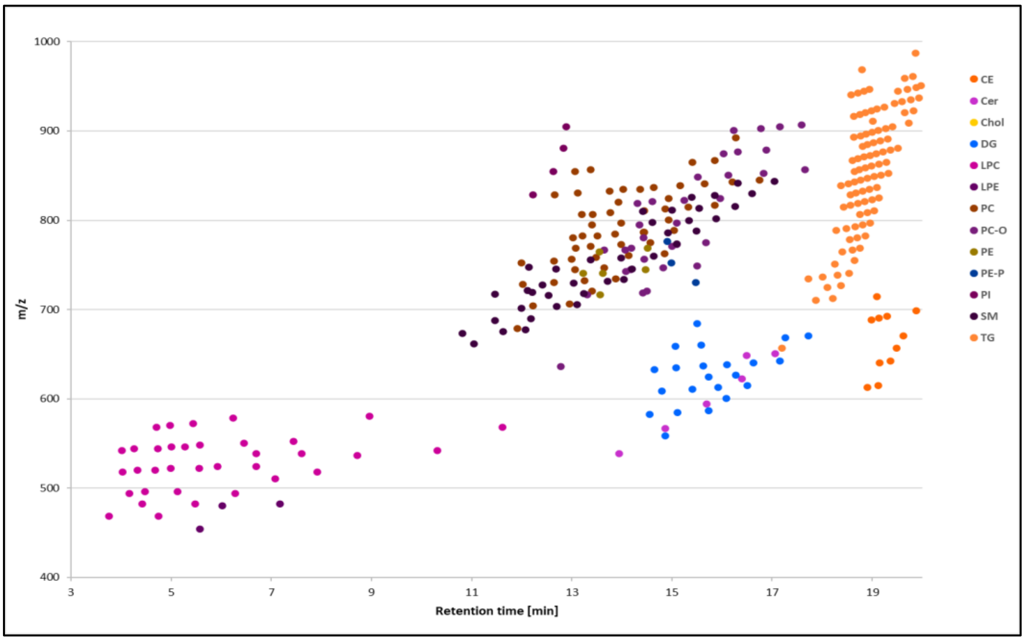

Supplementary Figure 2. Distribution of lipid features. The 2D plots show RT as a function of *m/z* for lipid species observed in negative (**a**) and positive (**b**) ion mode. Various lipid classes are indicated by different colors displayed as a legend for each sub-panel. Source data are provided as a Source Data file.

Supplementary Figure 3

a

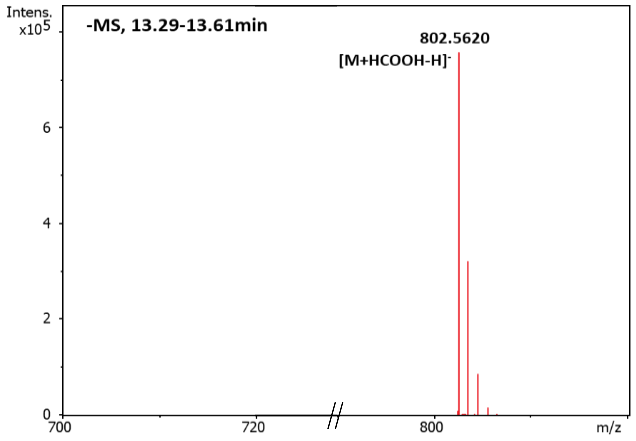

b

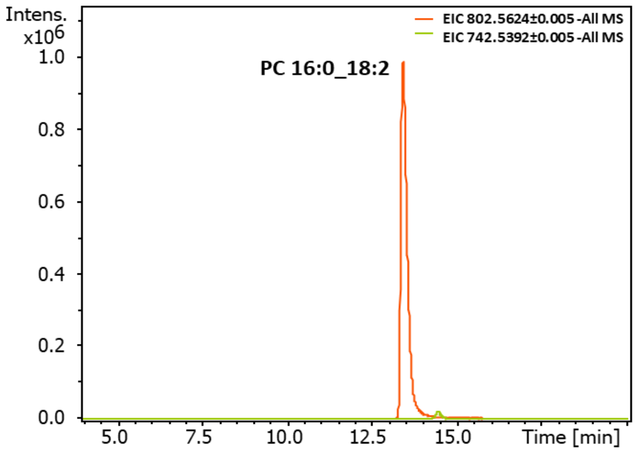

Supplementary Figure 3. In source fragmentation for phosphatidylcholine (PC). PCs are prone to in-source fragmentation depending on the analytical conditions. The mass spectrum (**a**) and the corresponding extracted ion chromatograms (EICs) of *m/z* 802.5620 [M+HCOOH-H]$^-$ and of *m/z* 742.5392 (**b**) show no formation of demethylated analog of PC 16:0_18:2 in NIST human plasma standard reference material (SRM) under the given analytical conditions in this study.

Supplementary Figure 4

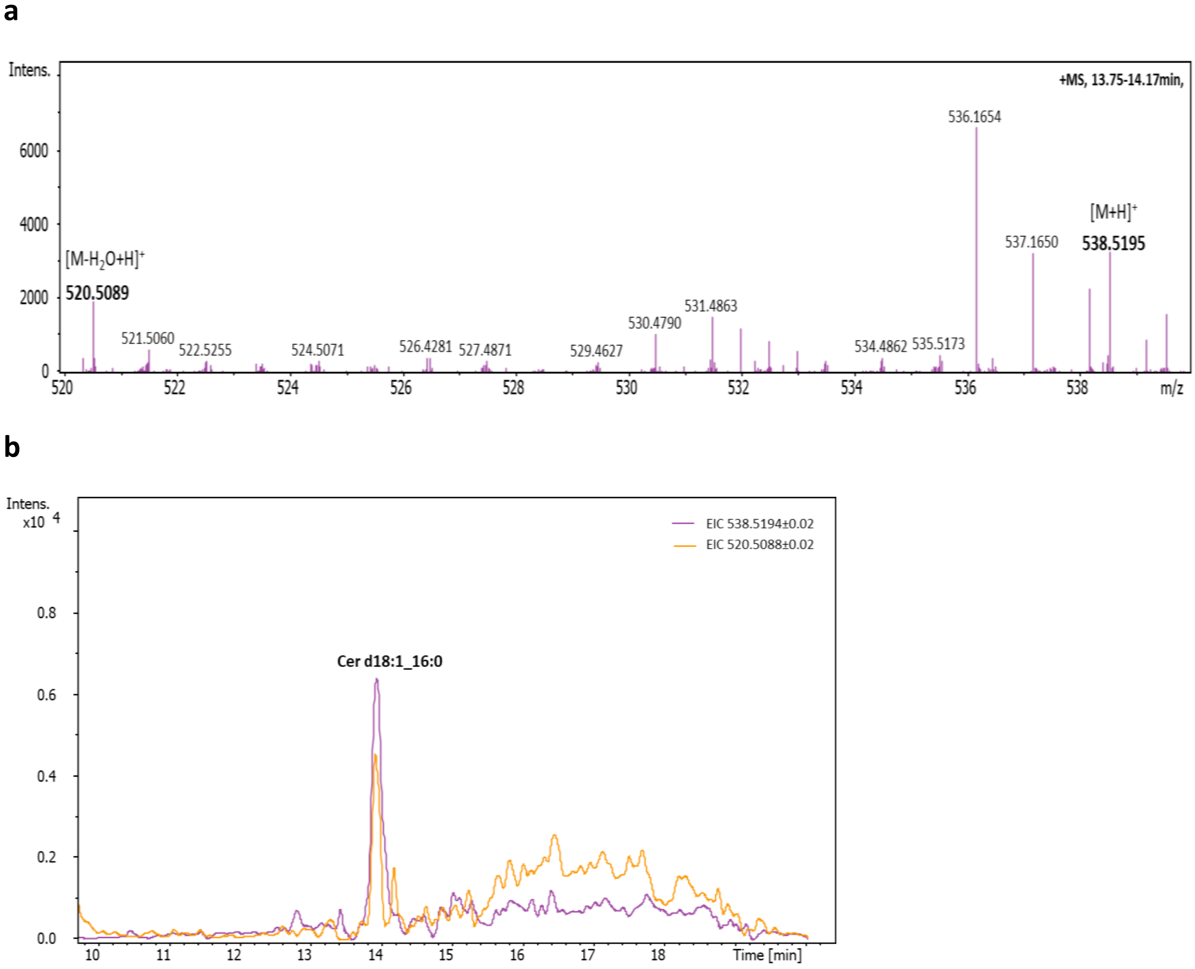

Supplementary Figure 4. In Source fragmentation for Ceramide (Cer). Ceramides are prone to in-source fragmentation depending on the analytical conditions. The mass spectrum of Cer d18:1_16:0 (**a**) and the corresponding extracted ion chromatograms (EICs) of *m/z* 538.5194 $[M+H]^+$ and of *m/z* 520.5088 $[M-H_2O+H]^+$ (**b**), respectively, reveal no loss of water for Cer d18:1_16:0 in NIST human plasma standard reference material (SRM) under the given analytical conditions in this study.

Supplementary Figure 5

a

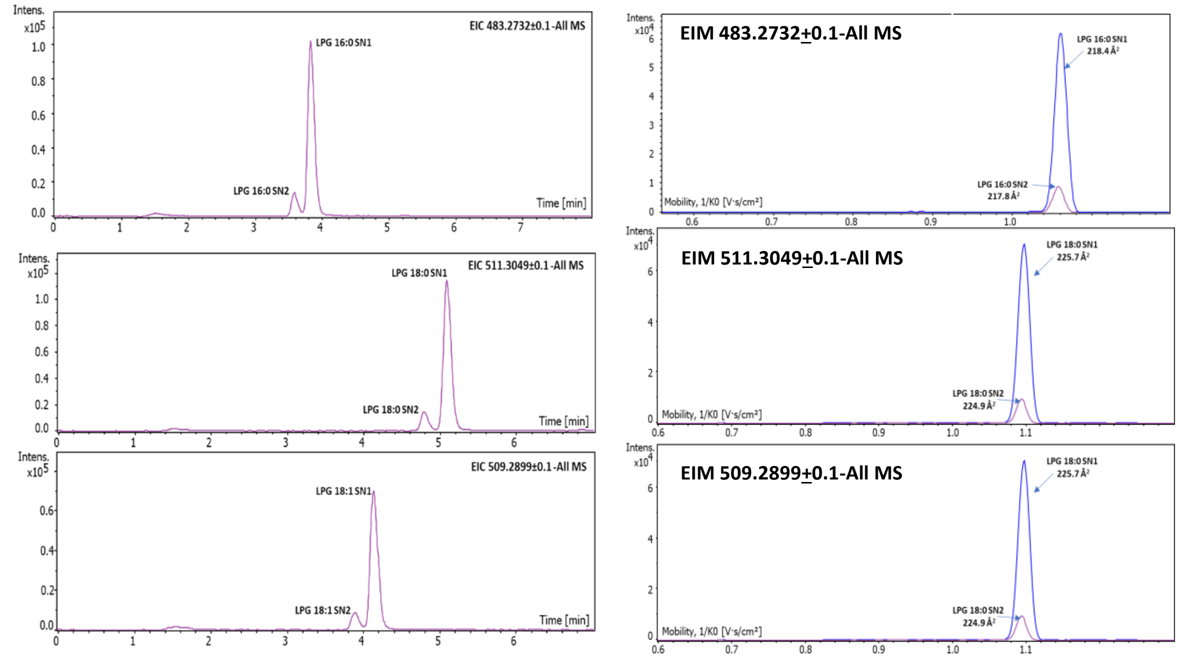

b

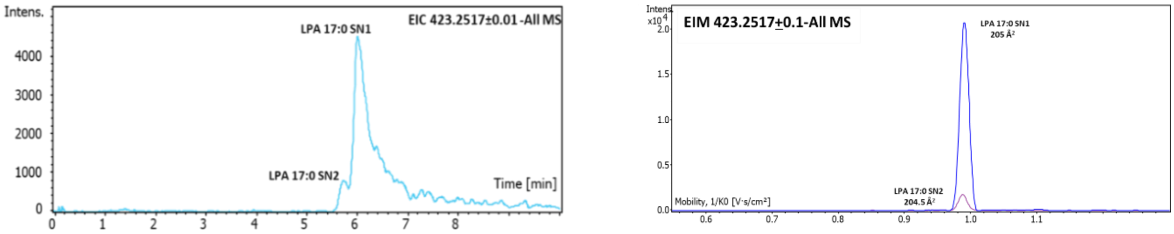

c

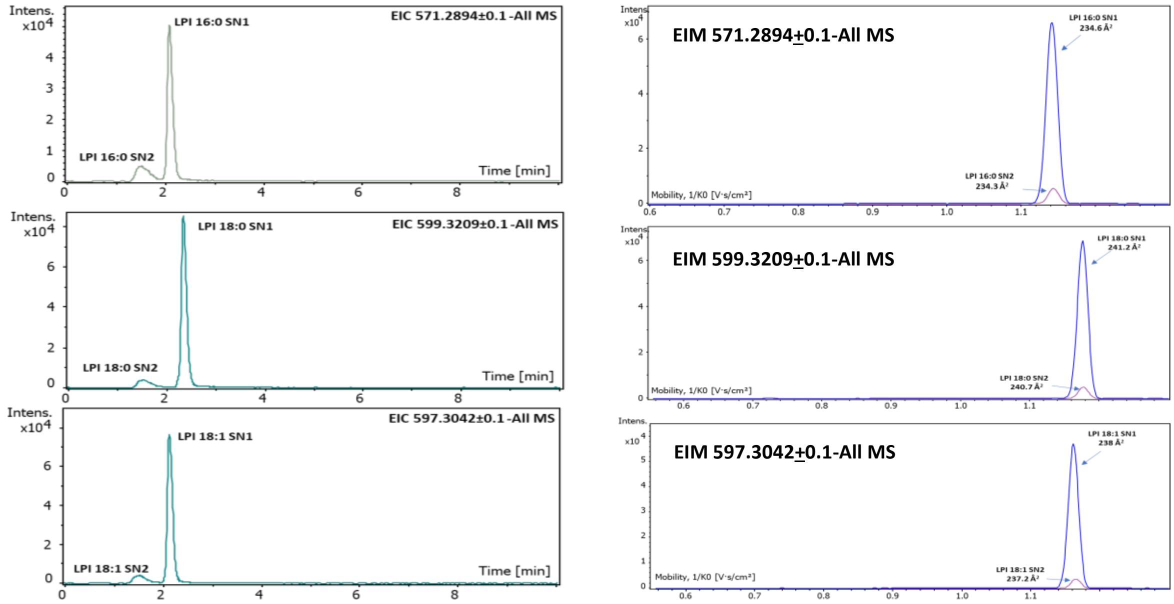

Supplementary Figure 5. SN1/SN2 separation of lysophospholipids. Chromatographic and mobilographic separation of SN1/SN2 isomers for lysophosphatidylglycerol (LPG): LPG 16:0, LPG 18:0, LPG 18:1 (**a**), lysophosphatidic acid (LPA): LPA 17:0 (**b**), and lysophosphatidylinositol (LPI): LPI 16:0, LPI 18:0, LPI 18:1 (**c**) lipid species.

Supplementary Figure 6

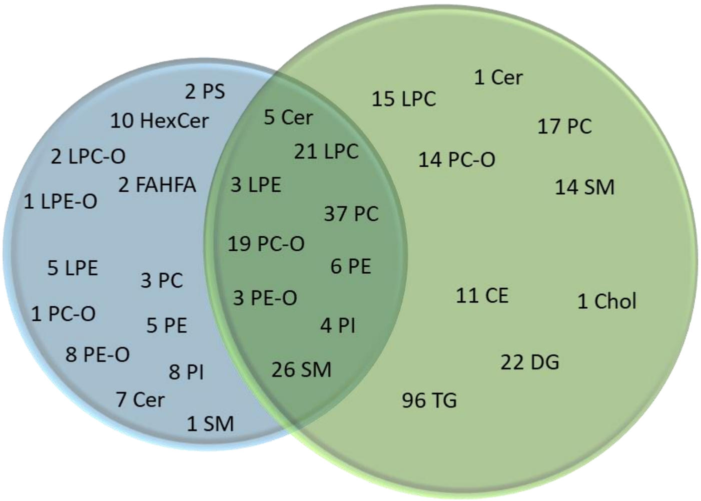

Supplementary Figure 6. Identified lipid species in different ion modes. Overall comparison of the number of unique and common (125) lipid species in each lipid class between negative-(blue) (55) and positive ion mode (green) (191). Source data are provided in Supplementary Data 6.

Supplementary Figure 7

a

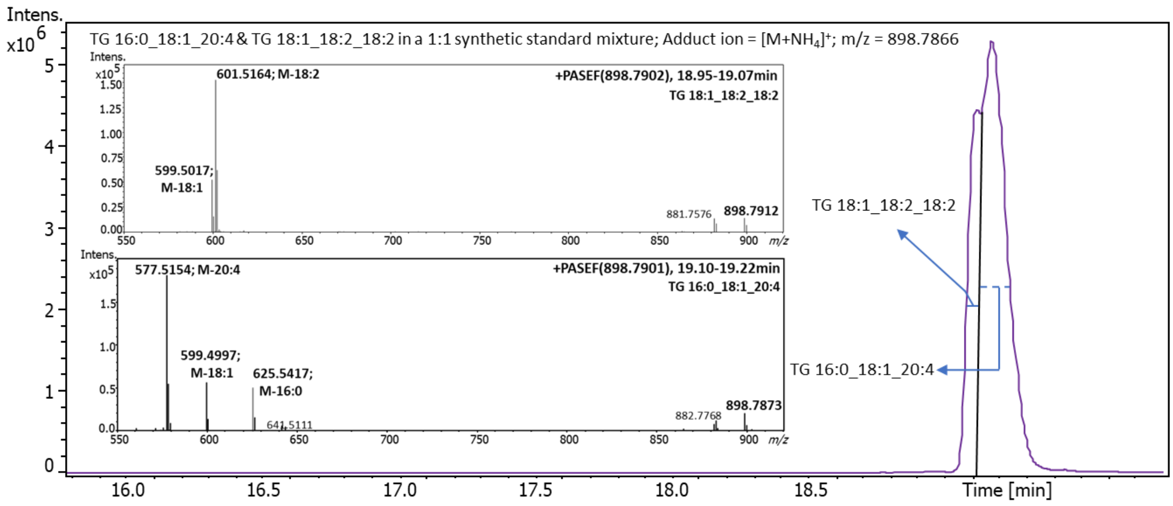

b

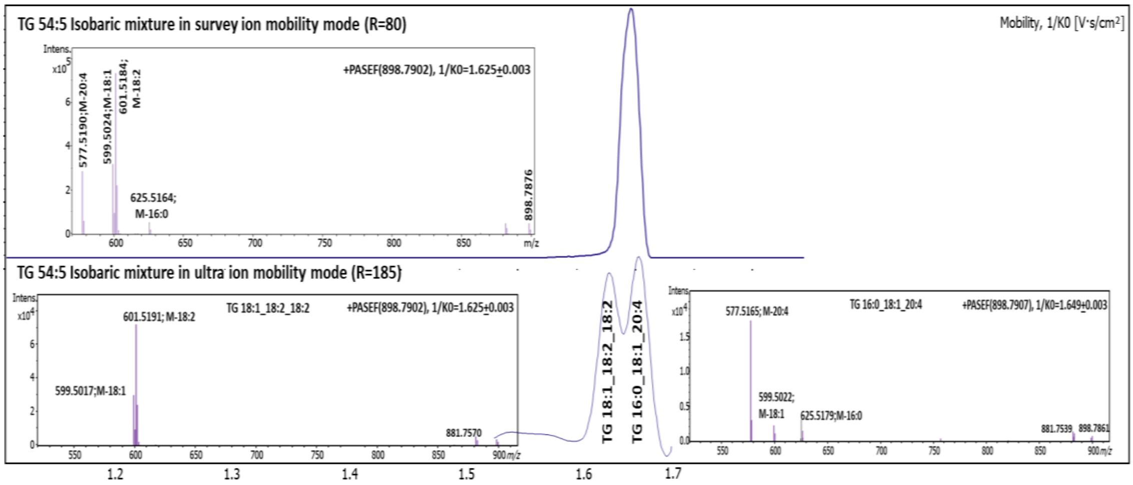

c

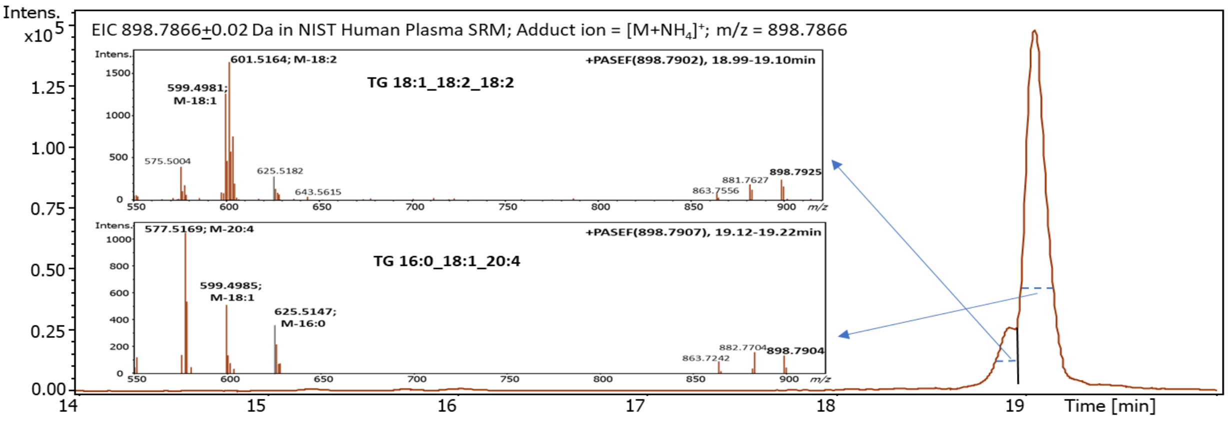

d

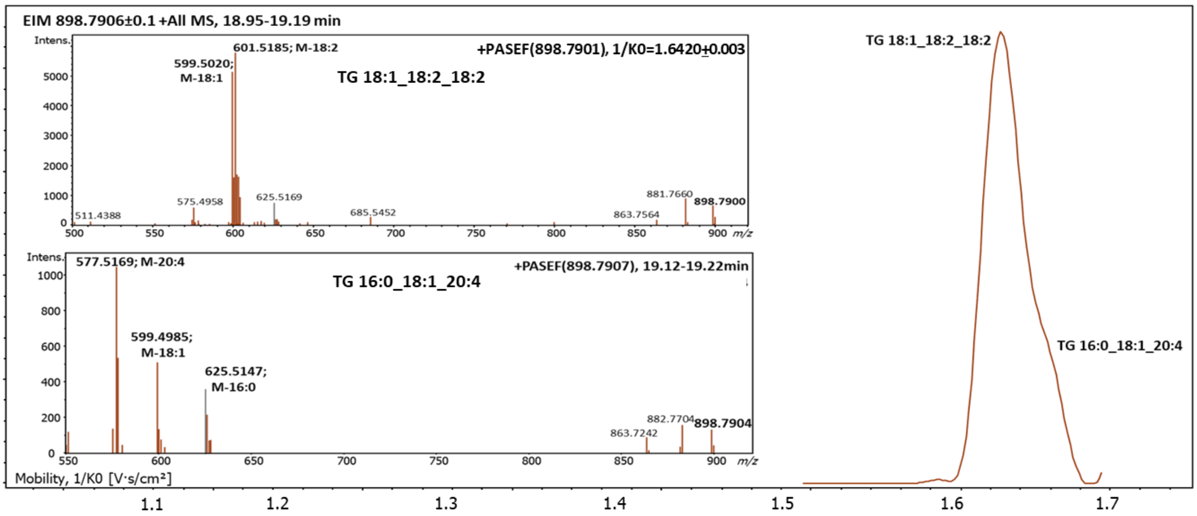

Supplementary Figure 7. Triglyceride (TG) Isomers in a 1:1 synthetic standard mixture and NIST human plasma standard reference material (SRM). The figure shows the extracted ion chromatograms (EIC) (**a**) and extracted ion mobilograms (EIM) (**b**) of the TG isomers: TG 54:5 (TG 16:0_18:1_20:4 and TG 18:1_18:2_18:2) in 1:1 synthetic standard mixture as well as the EIC (**c**) and EIM (**d**) of the TG 54:5 isomers in NIST plasma SRM along with their MS^2 spectra. The TG 54:5 isomers in the 1:1 synthetic standard mixture are shown in both survey (resolution = 80) and ultra (resolution = 185) ion mobility resolution mode (**b**) whereas for NIST plasma SRM only in ultra-ion mobility resolution (**d**). The MS parameters used for survey resolution mode are as described previously in the materials and method section whereas, for the ultra-resolution mode, the inverse ion mobility range was set between 1.50 to 1.90 $V.s/cm^2$.

Supplementary Figure 8

a

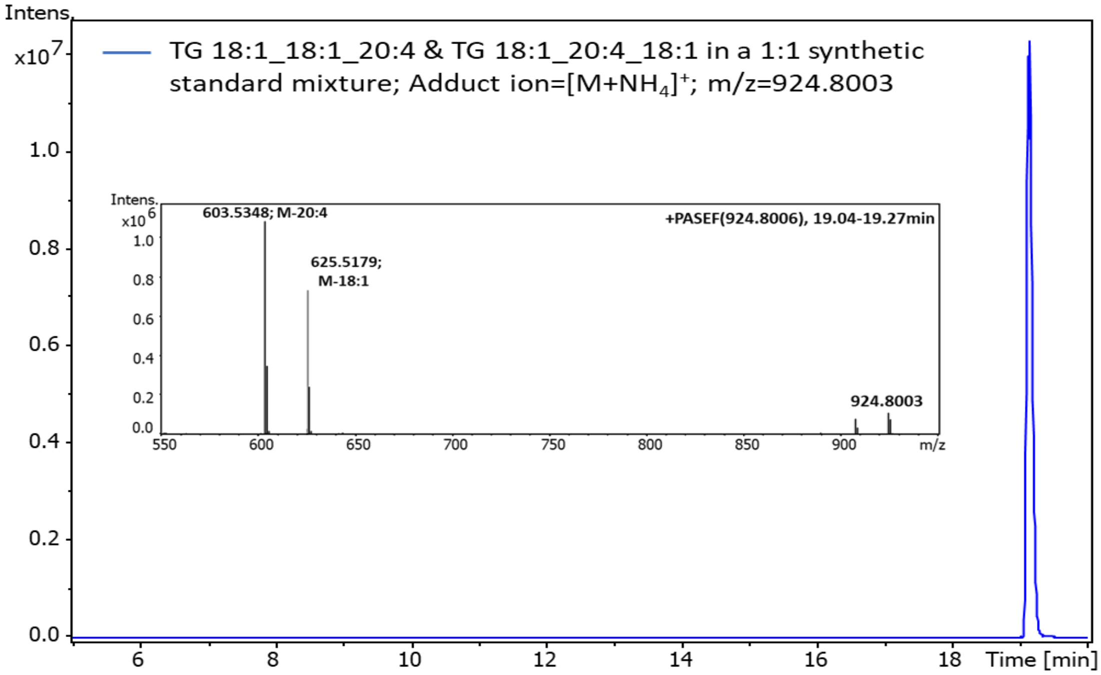

b

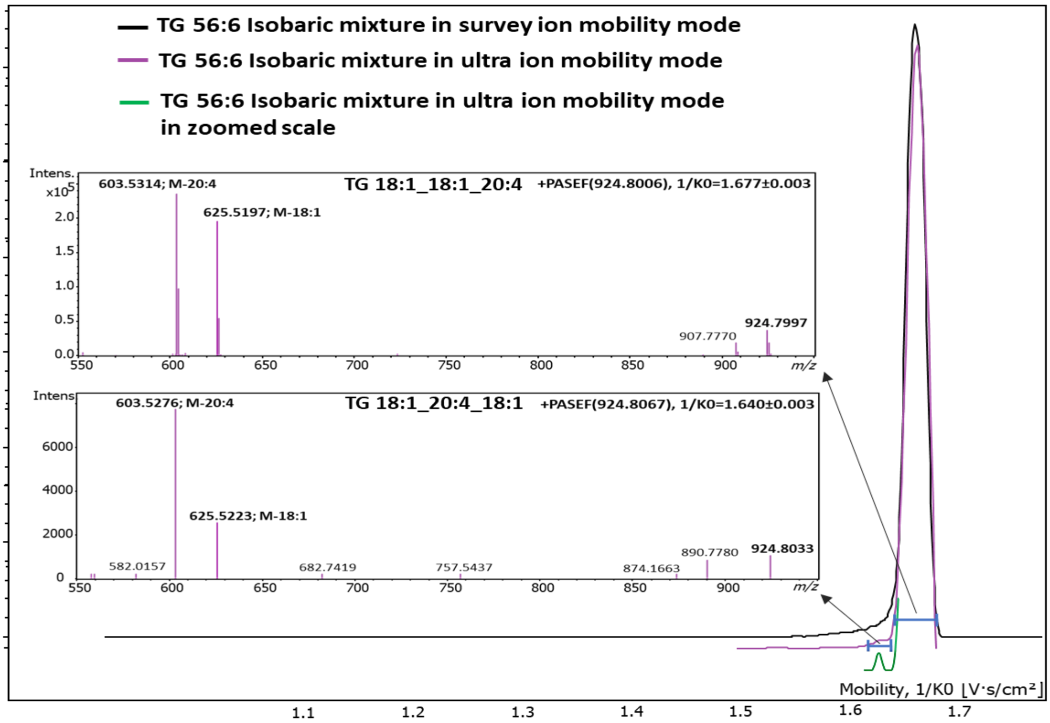

c

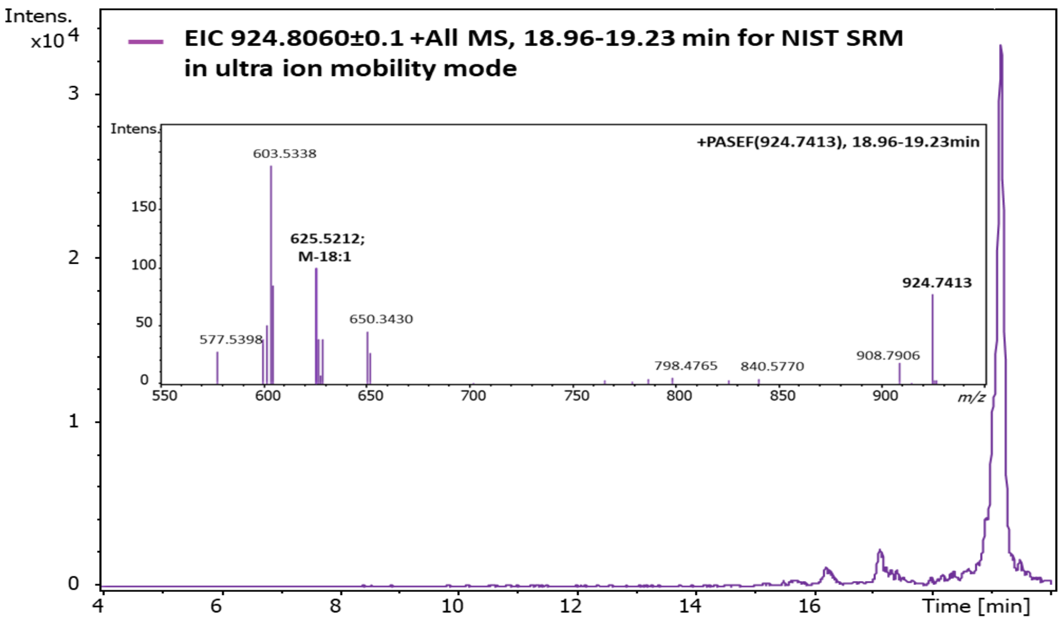

d

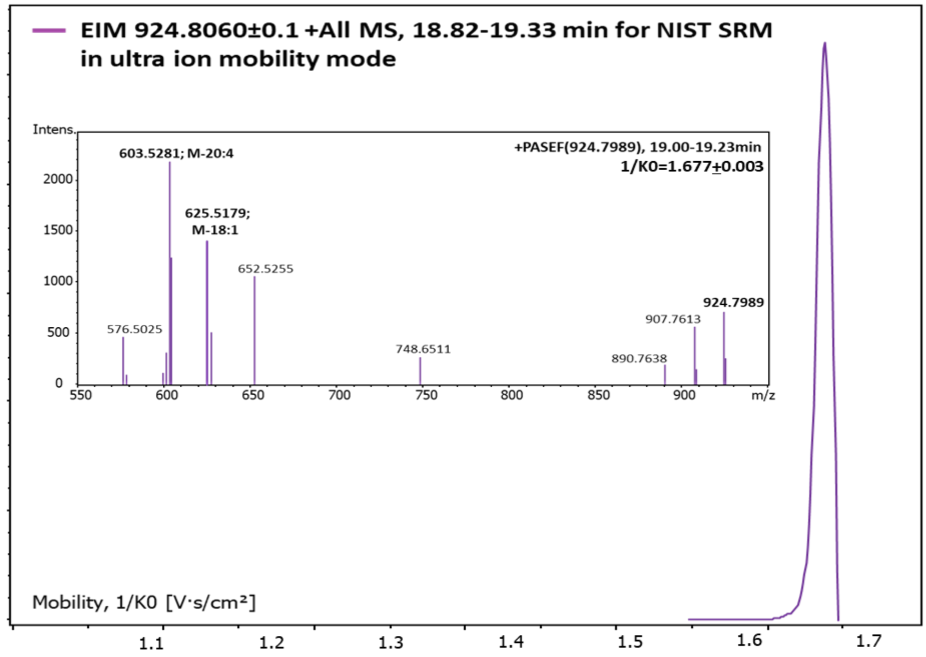

Supplementary Figure 8. Triglyceride (TG) Isomers in 1:1 synthetic standard mixture and NIST human plasma standard reference material (SRM). The figure shows the extracted ion chromatograms (EIC) (**a**) and extracted ion mobilograms (EIM) (**b**) of the TG isomers: TG 56:6 (TG 18:1_20:4_18:1 and TG 18:1_18:1_20:4) in 1:1 synthetic standard mixture as well as the EIC (**c**) and EIM (**d**) of the TG isomers in NIST plasma SRM a with their MS^2 spectra. The TG 56:6 isomers in the 1:1 synthetic standard mixture are shown in both survey (resolution = 80) and ultra (resolution = 185) ion mobility resolution mode (**b**) whereas only in ultra-ion mobility resolution for NIST plasma SRM (**d**). The MS parameters used for survey resolution mode are as described previously in the materials and method section whereas, for the ultra-resolution mode, the inverse ion mobility range was set between 1.50 to 1.90 V.s/cm^2.

Supplementary Figure 9

a

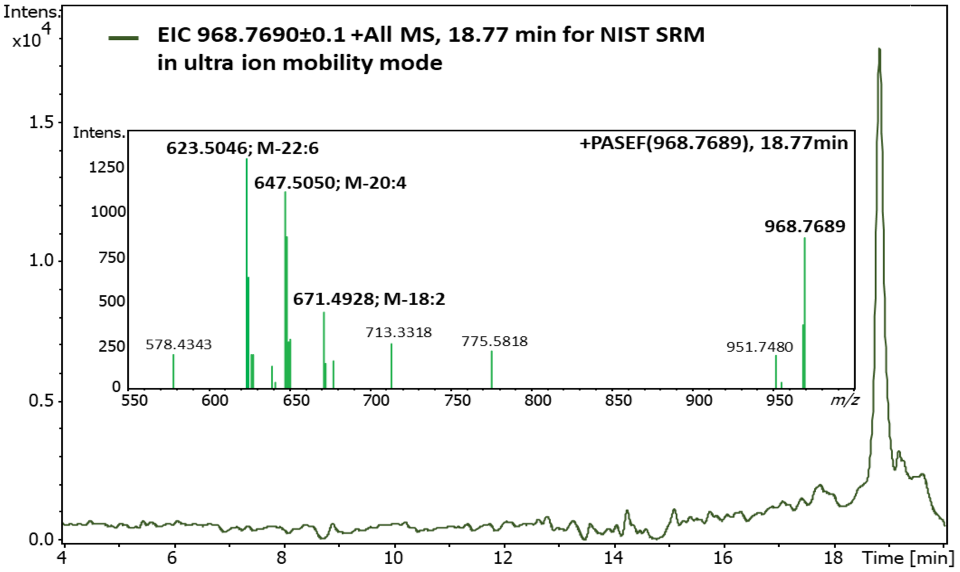

b

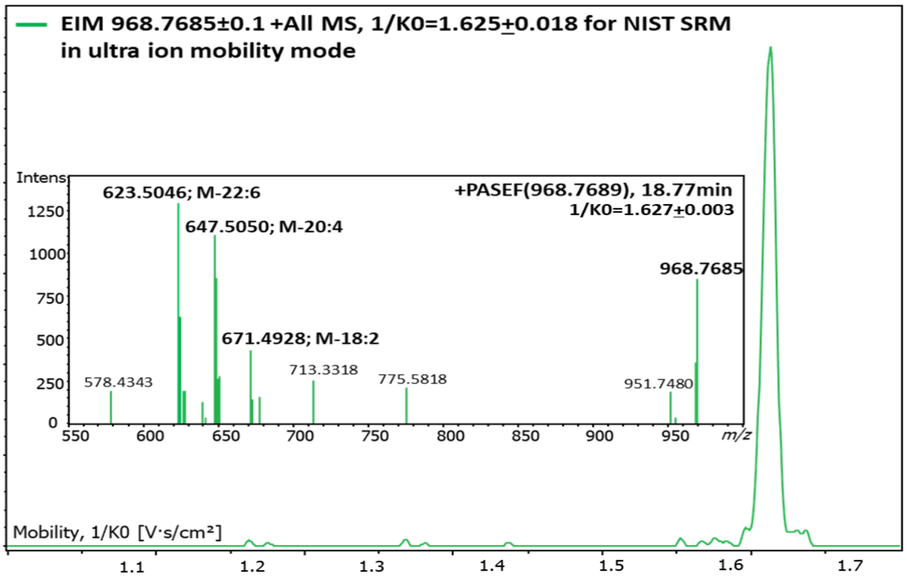

Supplementary Figure 9. Triglyceride (TG) Isomers in NIST human plasma standard reference material (SRM). The figure shows the extracted ion chromatograms (EIC) (**a**) and extracted ion mobilograms (EIM) (**b**) of the TG isomers: TG 60:12 (TG 18:2_20:4_22:6 and TG 20:4_20:4_20:4) in NIST plasma SRM together with their MS^2 spectra. The TG 60:12 isomeric mixture in NIST plasma SRM was analyzed in ultra (resolution = 185) ion mobility resolution mode. The inverse ion mobility range for the ultra-resolution mode was set between 1.50 to 1.90 V.s/cm2.

Supplementary Figure 10

a

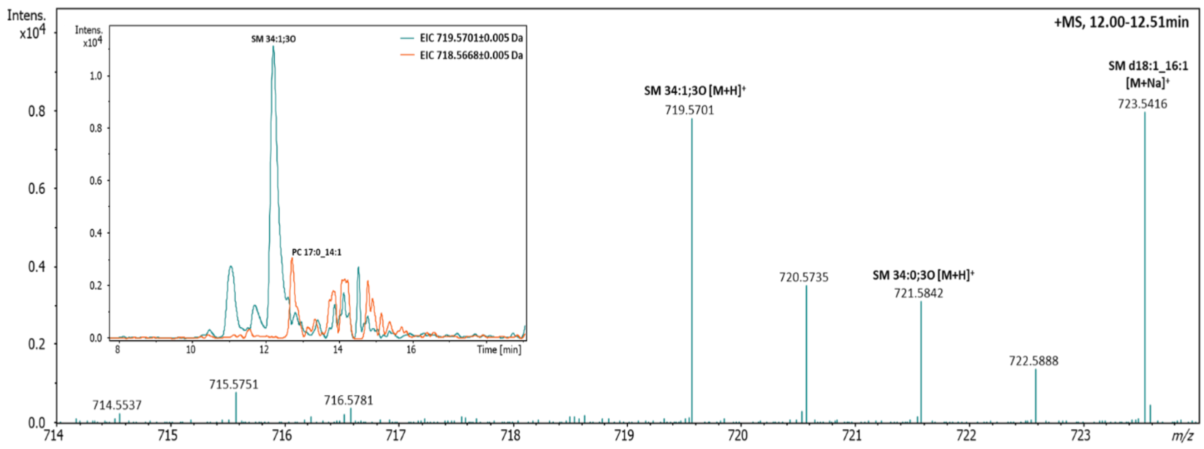

b

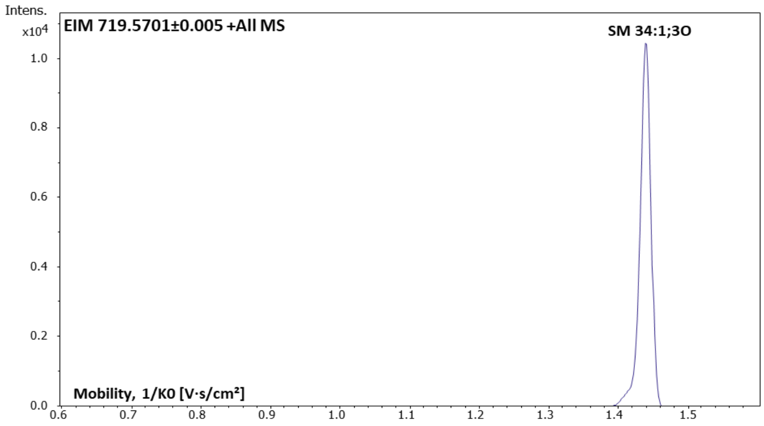

Supplementary Figure. 10 Isotope interference. Extracted ion chromatogram (EIC) (**a**) for the monoisotopic peak of sphingomyelin (SM) SM 34:1;3O (*m/z* 719.5701 Da) and for the peak at *m/z* 718.5668 corresponding to 1.00335 Da less than the monoisotopic peak of SM 34:1;3O and extracted ion mobilogram (EIM) (**b**) for the monoisotopic peak of SM 34:1;3O ($1/K_0$=1.425 V.s/cm^2). No overlapping for the two EIC suggests no isotope interference for SM 34:1;3O precursor arising from a possible isotopic peak of phosphatidylcholine (PC) PC 17:0_14:1. This is also confirmed by the absence of *m/z* 718.5668+0.02 Da in the survey TOF MS scan for that particular chromatographic region of interest.

Supplementary Figure 11

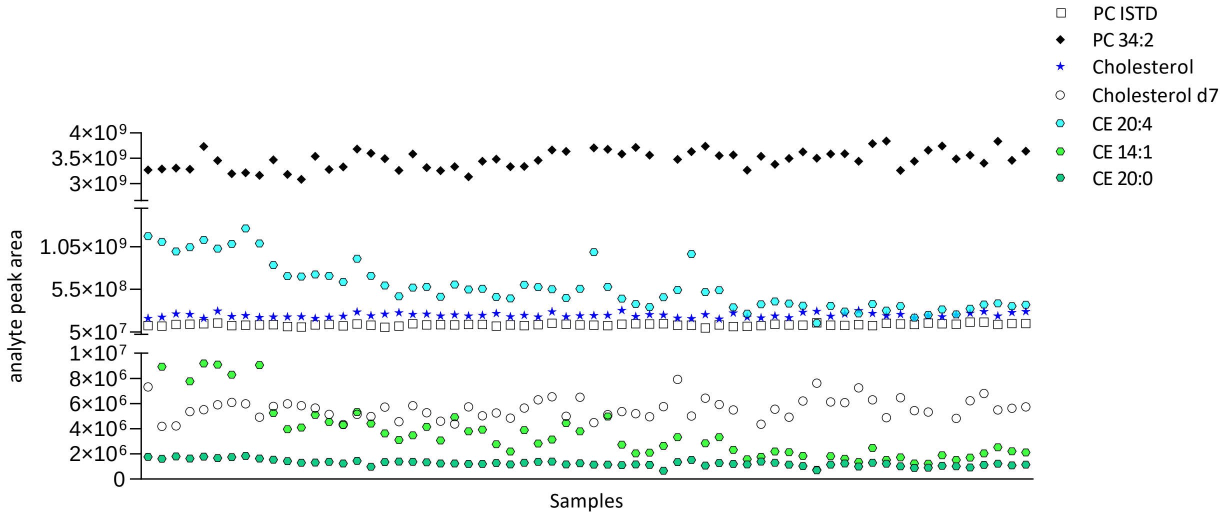

Supplementary Figure 11. Variation in analyte peak areas of representative cholesterol esters (CE), phosphatidylcholines (PC), and cholesterol species. The figure shows a plot of analyte peak area vs sample position for CE, cholesterol, and PC. The plot shows the decrease in analyte peak area of hydrophobic CE species, especially for medium to high abundant molecules in relation to their corresponding internal standard (ISTD), used for quantification. Source data are provided in Supplementary Data 7.

Supplementary Figure 12

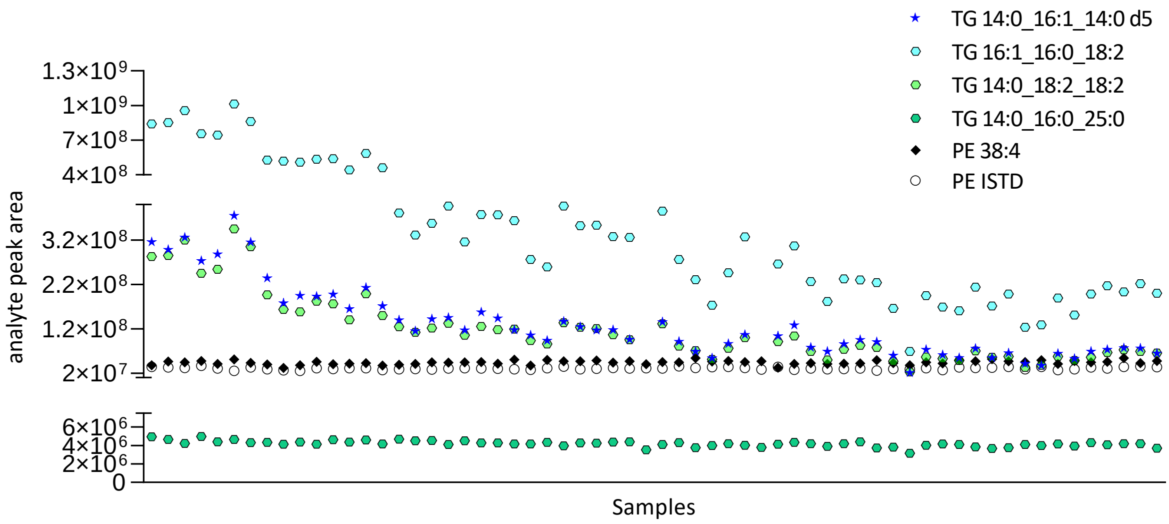

Supplementary Figure 12. Variation in analyte peak areas of triglycerides (TG) and phosphatidylethanolamines (PE). The figure shows a plot of the analyte peak area vs sample position for TG and PE species. The plot shows the decrease in the analyte peak area of hydrophobic TG species, especially for medium to high abundant molecules in relation to their corresponding internal standard (ISTD), used for quantification. Source data are provided in Supplementary Data 7.

Supplementary Figure 13

a

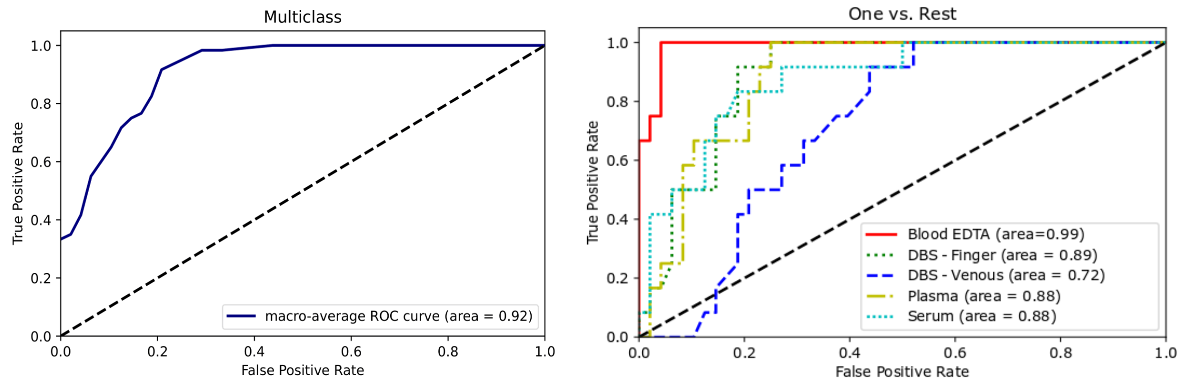

b

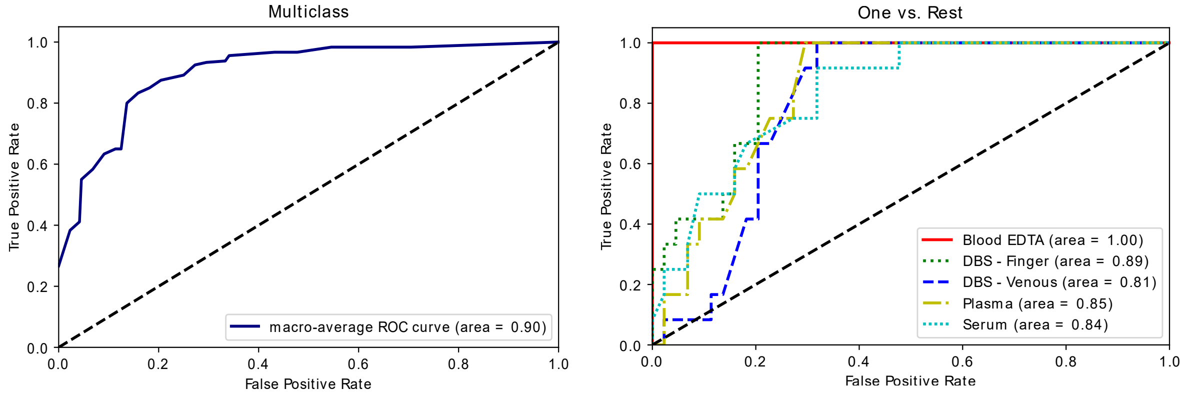

Supplementary Figure 13. Statistical output for lipid classifiers derived from the LBlooD study. The multiclass classification results are shown as a plot of true vs false positive rate in neg ion mode (**a**) and pos ion mode (**b**). The true vs false positive rate with the AUROC value for each biological matrix in a one vs rest classification are shown in neg ion mode (**a**) and pos ion mode (**b**). Source data are provided as a Source Data file.

Supplementary Figure 14

a

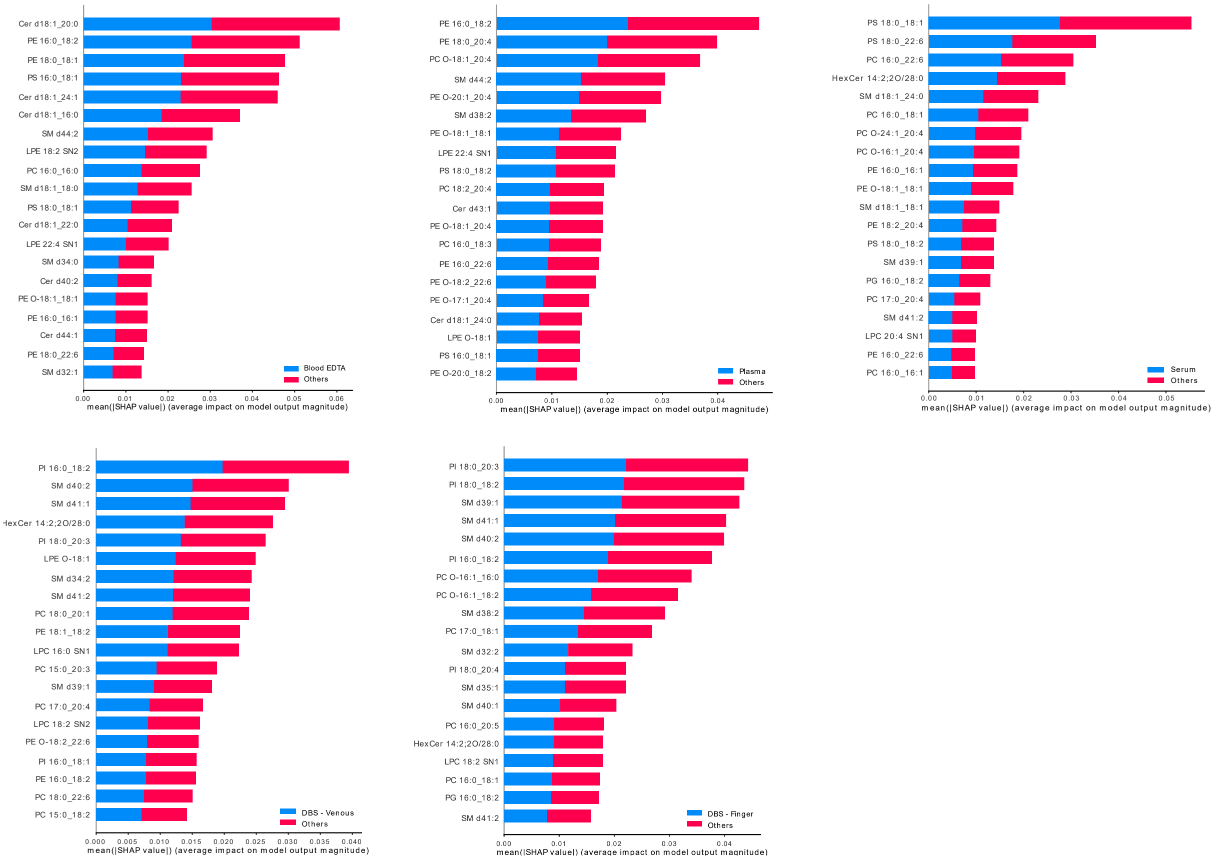

b

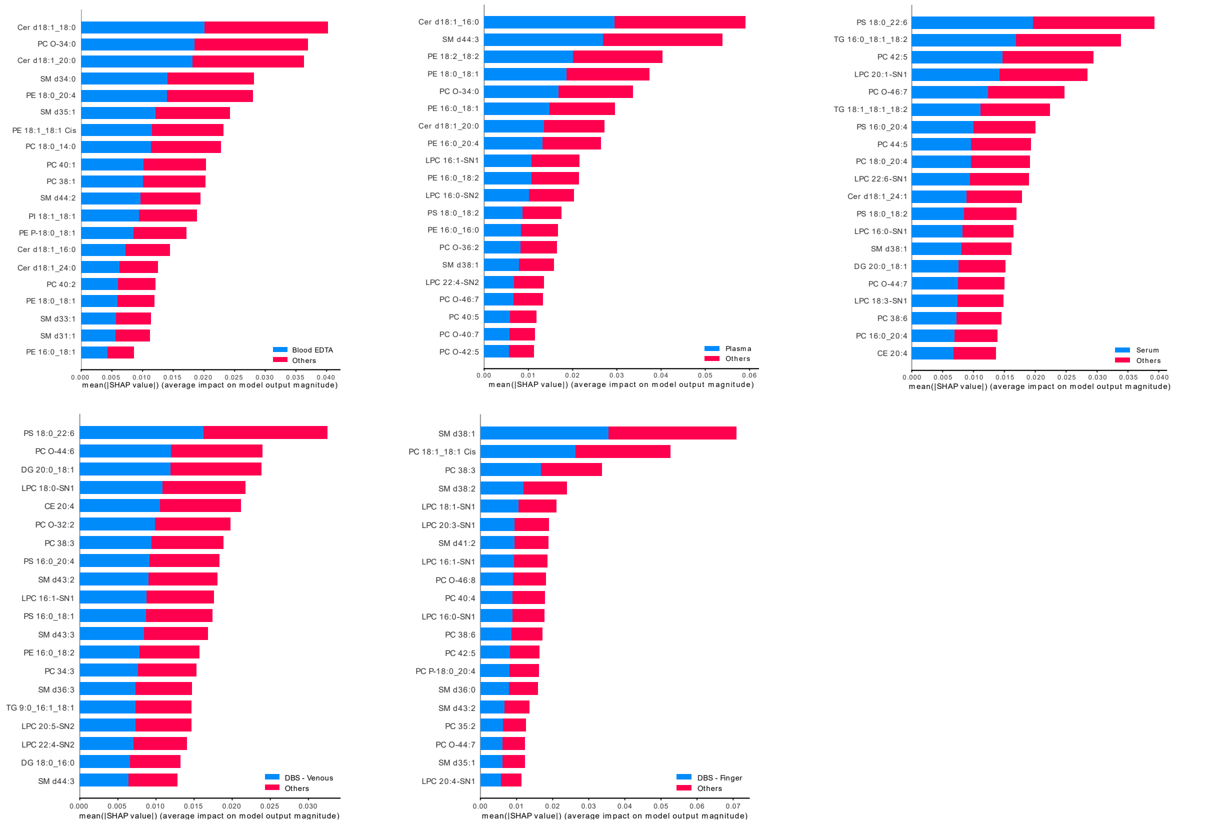

Supplementary Figure 14. Statistical output for lipid classifiers derived from the LBlooD study. Representation of the mean SHAP value of the characteristic lipid species for one biological matrix compared to the rest resulting from the one vs rest classification in neg ion mode (**a**) and pos ion mode (**b**). Source data are provided as a Source Data file.

Supplementary Figure 15

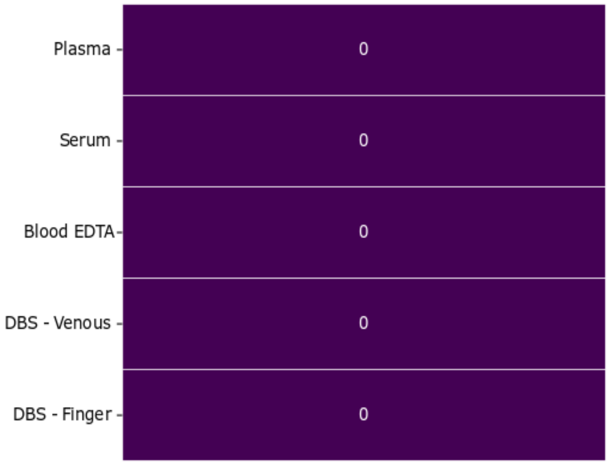

Timepoint variation – Friedman test

Supplementary Figure 15. LBlooD study timepoint analysis for all 4 persons. Visualization of Friedman test showing the time-point variation in the concentration of identified lipid species merged for all four persons. H=0 from the plot indicates no significant difference between the time points for each biological matrix. Source data are provided as a Source Data file.

Supplementary Figure 16

a

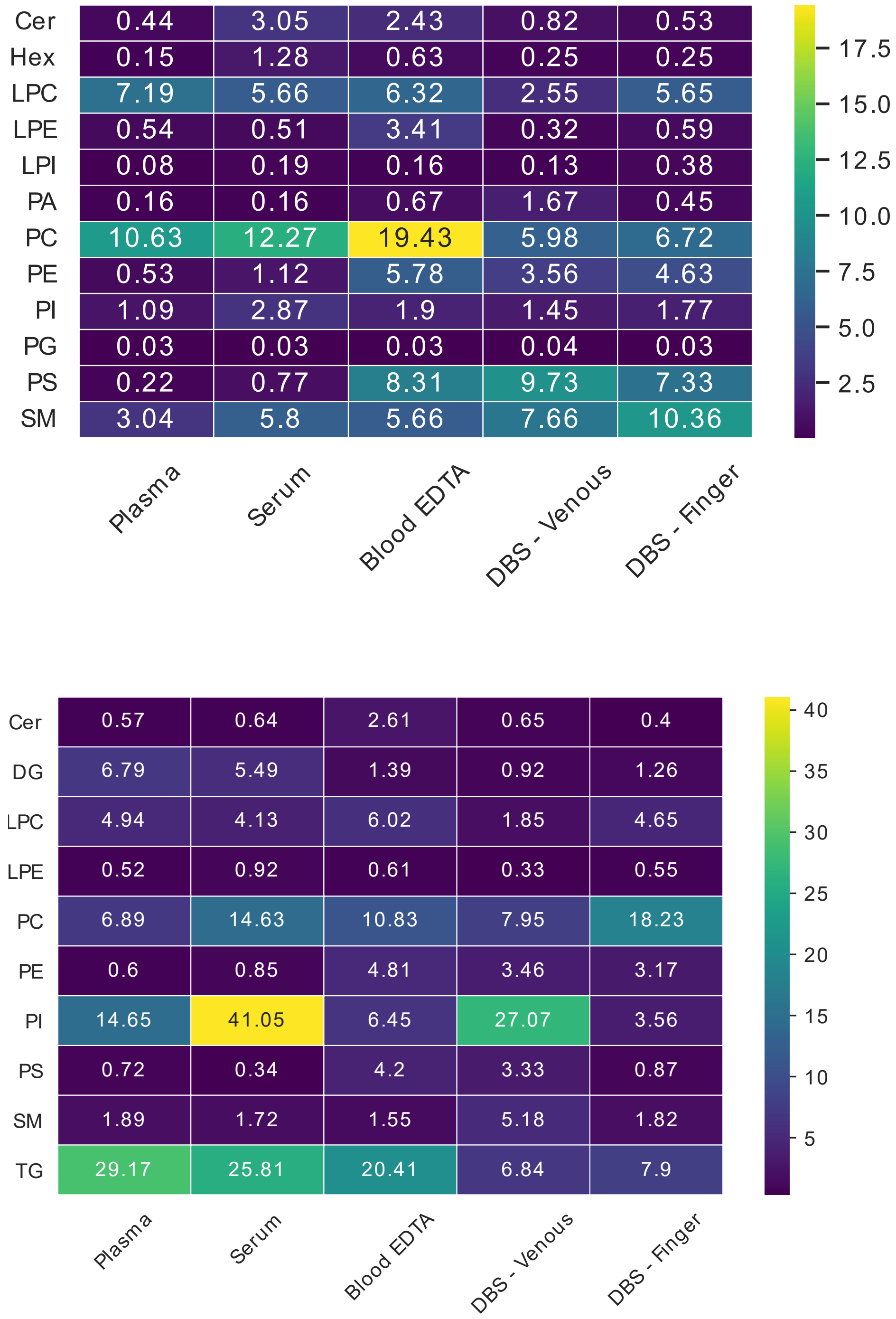

Supplementary Figure 16. Lipid class variation over time. The figure shows the variation of each lipid class over the three-time points in both negative (**a**) and positive (**b**) mode analysis for one of the participating individuals. The color bar in the scale represents the standard deviation (SD) range with violet representing low SD and yellow representing high SD. Source data are provided as a Source Data file.

Supplementary Figure 17

a

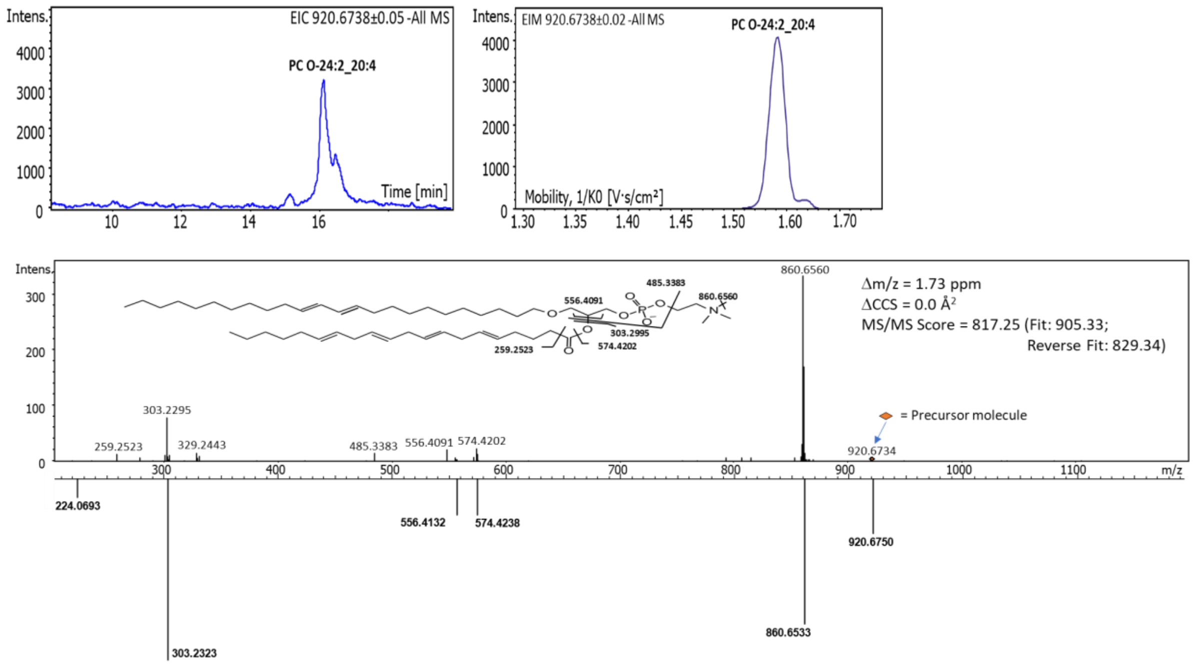

b

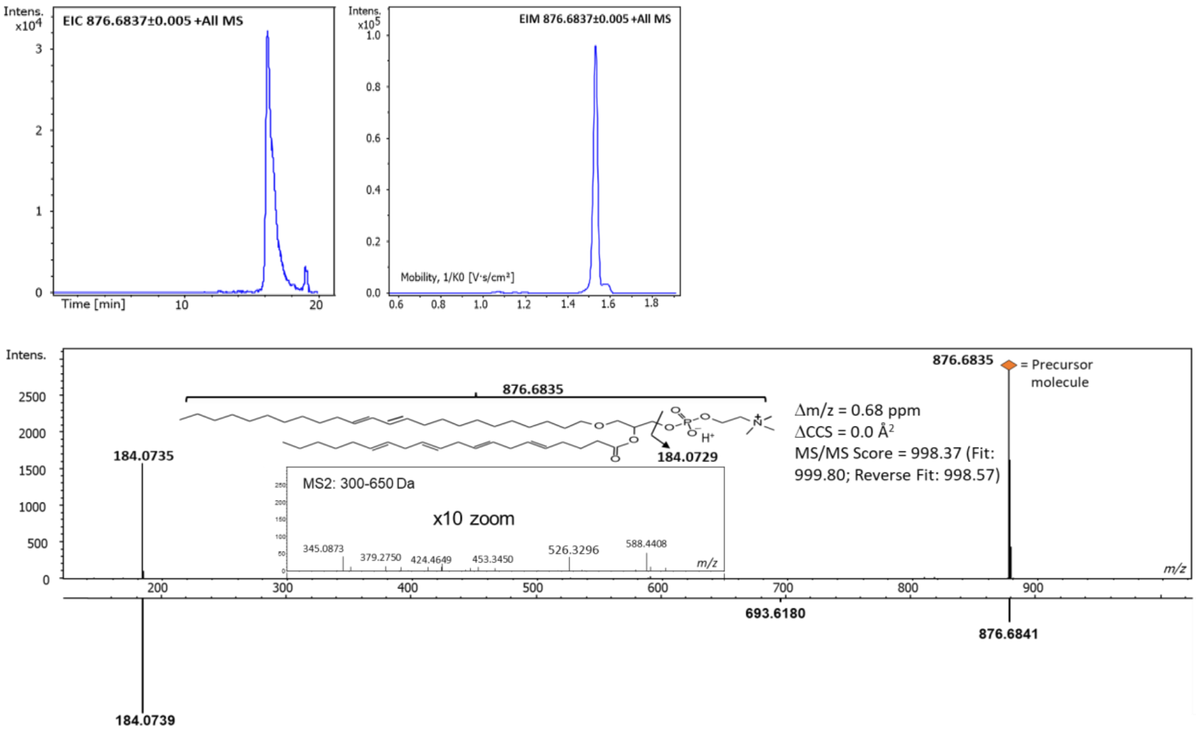

Supplementary Figure 17. In-house library generation - phosphatidylcholine (PC) PC O-24:2_20:4. Manual curation of the MS^2 Spectrum: The structural characterization of PC O-24:2_20:4 in negative (**a**) and positive (**b**) modes using their respective extracted ion chromatogram (EIC) and extracted ion mobilogram (EIM). The structural fragments expected by fragmentation of the same molecule in each polarity are shown by cleavages in the structure. The figure also shows the collinearity between the fragments from their parallel accumulation serial fragmentation (PASEF) Scan and MS-DIAL spectral library[3].

Supplementary Figure 18

a

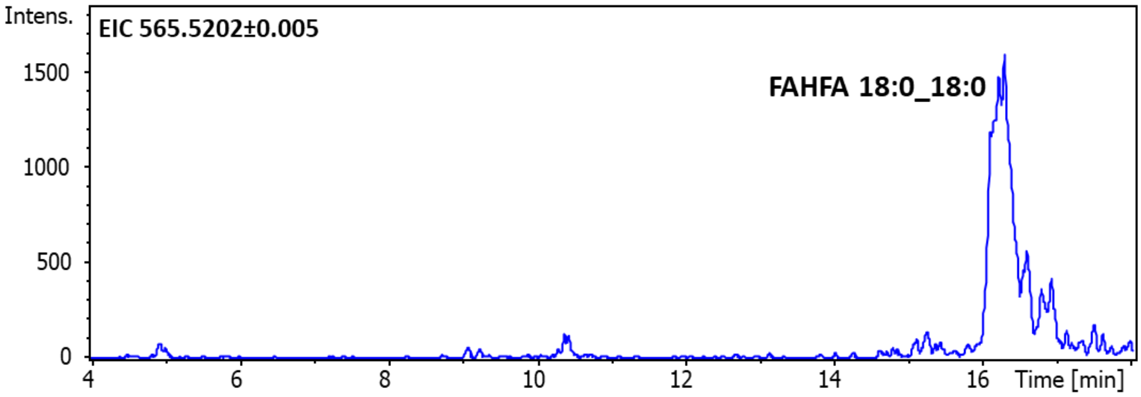

b

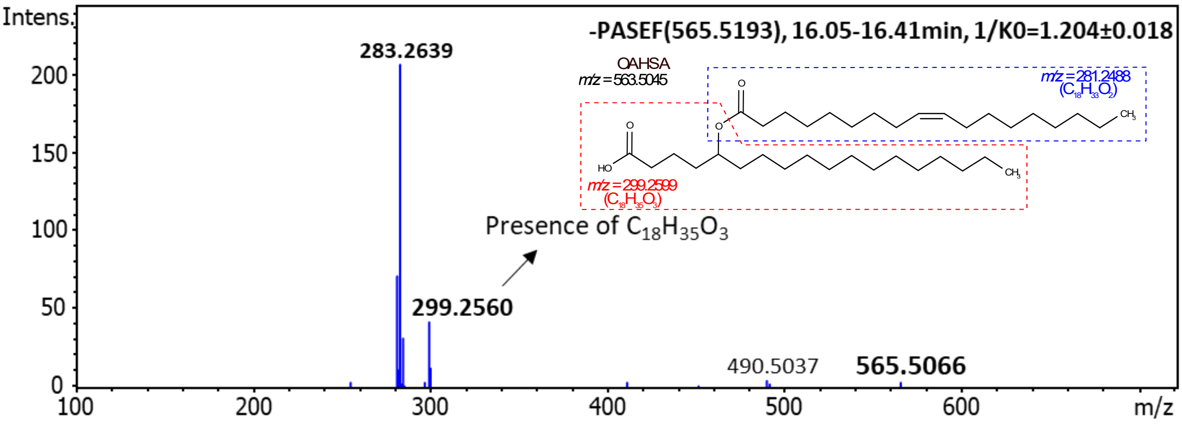

Supplementary Figure 18. In-house library generation - fatty acid esters of hydroxy fatty acids (FAHFA) FAHFA 18:0_18:0. Manual curation of the MS^2 Spectrum: the figure shows the extracted ion chromatogram (EIC) (**a**) and MS^2 of the monoisotopic peak of FAHFA 18:0_18:0 (**b**) from the measurements of the synthetic standard of FAHFA 18:0_18:0 which was used to generate the in-house library. Additionally, the presence of diagnostic ions at *m/z* 299.2560 in the parallel accumulation serial fragmentation scan of FAHFA 18:0_18:0 indicates the presence of FAHFA analyte and not of a dimer of fatty acid (FA) FA 18:0 (Nelson et al.)[5].

Supplementary Figure 19

a

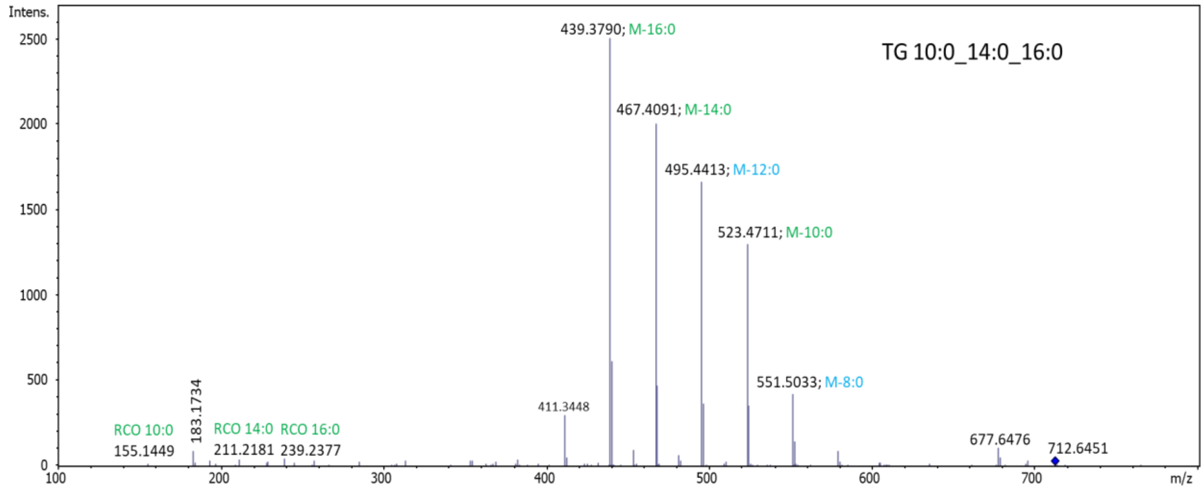

b

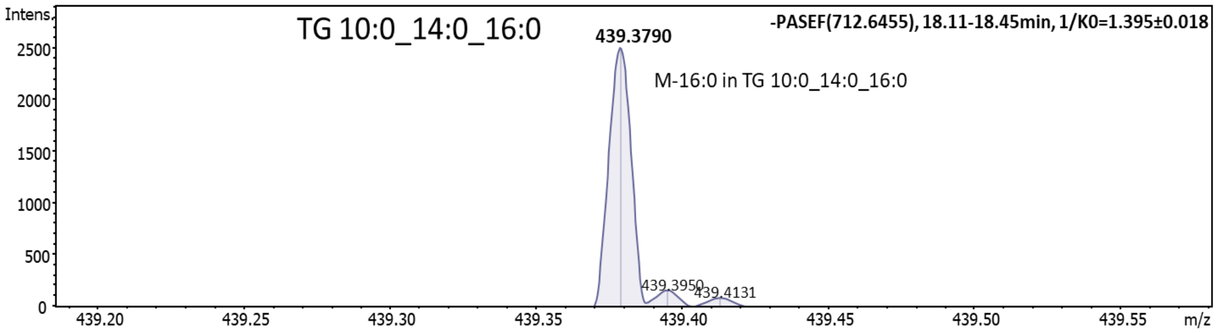

Supplementary Figure 19. In-house library generation - triglyceride (TG) TG 10:0_14:0_16:0. Manual curation of the MS^2 spectrum: TG 10:0_14:0_16:0 in positive mode with their characteristic fragments (**a**) and zoomed-in profile MS^2 spectra of TG 10:0_14:0_16:0 around the fragment ions at *m/z* 439.3793 Da (**b**). The most abundant alkyl fragments corresponding to a single isobaric structure were inferred and used for feature annotation.

Supplementary Figure 20

a

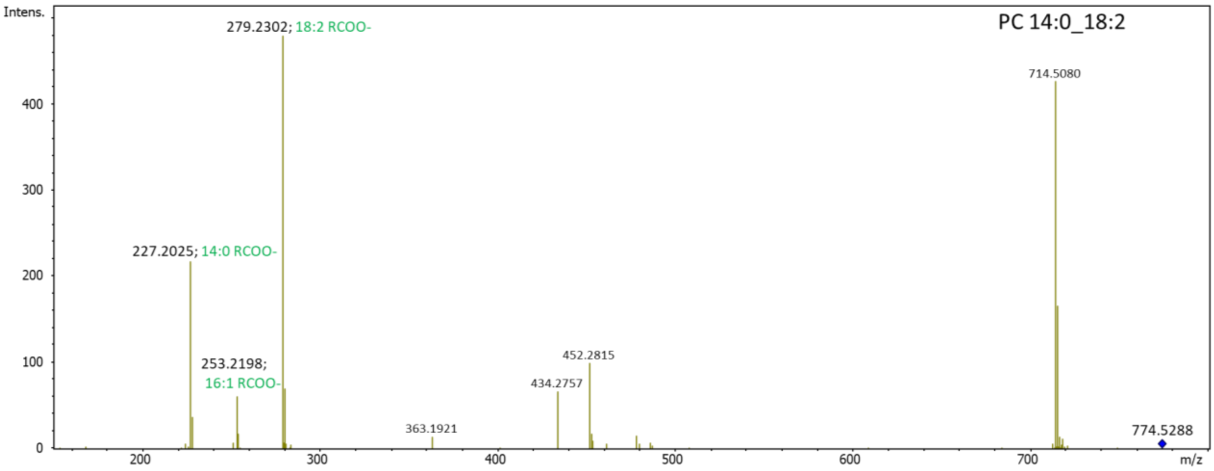

b

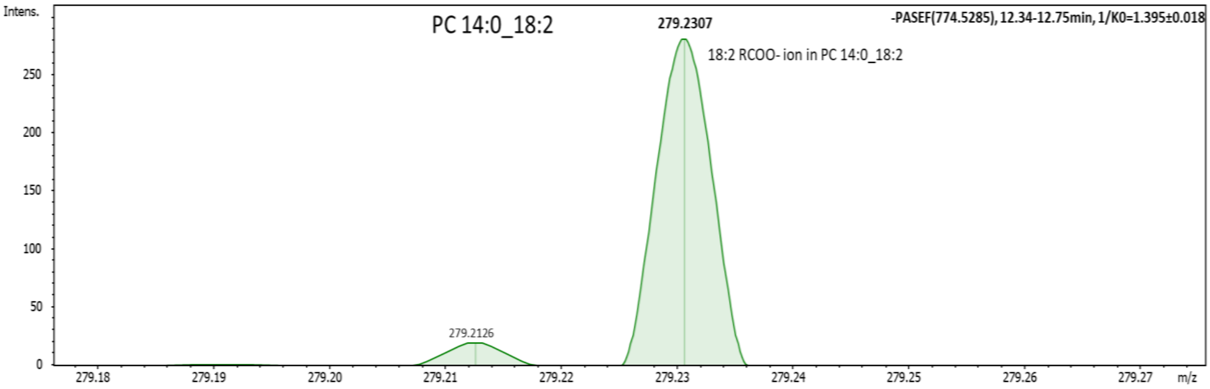

Supplementary Figure 20. In-house library generation – phosphatidylcholine (PC) PC 14:0_18:2. Manual curation of the MS^2 Spectrum: PC 14:0_18:2 in negative mode (**a**) and zoomed-in profile MS^2 spectra of PC 14:0_18:2 around the fragment ions at *m/z* 279.2307 (**b**).

Supplementary Table 1

negative ion mode

Source	In-house	CCS predict	Δ CCS with CCS predict	Vasilopoulou et al., 2020	Δ CCS with Vasilopoulou et al., 2020
Unit	(Å^2)	(Å^2)	(%)	(Å^2)	(%)
Cer ESTD	258.30	248.30	3.87	n.a.	n.a.
SM ESTD	285.90	280.80	1.78	n.a.	n.a.
PC ESTD	292.50	286.40	2.09	291.60	0.31
PE ESTD	269.10	265.00	1.52	268.40	0.26
PI ESTD	289.60	284.40	1.80	n.a.	n.a.
PG ESTD	275.50	269.30	2.25	n.a.	n.a.
PS ESTD	278.60	271.30	2.62	n.a.	n.a.

positive ion mode

Source	In-house	CCS predict	Δ CCS with CCS predict	Vasilopoulou et al., 2020	Δ CCS with Vasilopoulou et al., 2020
Unit	(Å^2)	(Å^2)	(%)	(Å^2)	(%)
Cer ESTD	258.10	256.20	0.74	n.a.	n.a.
SM ESTD	285.50	282.00	1.23	284.40	0.39
PC ESTD	290.30	288.10	0.76	291.30	0.34
PE ESTD	277.60	276.60	0.36	n.a.	n.a.
PI ESTD	299.50	294.70	1.60	n.a.	n.a.
PG ESTD	290.20	283.50	2.31	n.a.	n.a.
PS ESTD	285.20	280.80	1.54	n.a.	n.a.

Supplementary Table 1. Collision Cross Sections (CCS) comparison with CCS Predict. Comparison of in-house obtained CCS values (Å^2) with CCS predict[1] values and those reported by Vasilopoulou et al.[2] for negative and positive ion mode. Deviations of in-house obtained CCS values from CCS-predict values and those of Vasilopoulou et al. are given as ΔCCS in percentages (%), respectively.

Supplementary Table 2

MS DIAL 189 -10 double -> 179								
RT [...	m/z meas.	CCS (...	Name	Molecular For...	Ions	Annotations	AQ	Annotation Source
12.79	674.47660	259.7	LNAPE 14:1/N-17:0	$C_{36}H_{70}NO_8P$		SL		MSDIAL-TandemMassSpectralAtlas...
13.83	740.52327	271.7	LNAPE 18:2/N-18:1	$C_{41}H_{76}NO_8P$		SL		MSDIAL-TandemMassSpectralAtlas...
13.21	738.50742	269.5	LNAPE 18:2/N-18:2	$C_{41}H_{74}NO_8P$		SL		MSDIAL-TandemMassSpectralAtlas...
13.59	738.50794	271.0	LNAPE 20:4/N-16:0	$C_{41}H_{74}NO_8P$		SL		MSDIAL-TandemMassSpectralAtlas...
14.48	766.53920	277.4	LNAPE 20:4/N-18:0	$C_{43}H_{78}NO_8P$		SL		MSDIAL-TandemMassSpectralAtlas...
13.83	764.52322	275.7	LNAPE 20:4/N-18:1	$C_{43}H_{76}NO_8P$		SL		MSDIAL-TandemMassSpectralAtlas...
13.56	714.50781	267.0	PE 16:0_18:2	$C_{39}H_{74}NO_8P$		SL		MSDIAL-TandemMassSpectralAtlas...
13.52	762.50775	275.3	PE 16:0_22:6	$C_{43}H_{74}NO_8P$		SL		MSDIAL-TandemMassSpectralAtlas...
14.45	742.53912	273.5	PE 18:0_18:2	$C_{41}H_{78}NO_8P$		SL		MSDIAL-TandemMassSpectralAtlas...
14.41	790.53917	281.7	PE 18:0_22:6	$C_{45}H_{78}NO_8P$		SL		MSDIAL-TandemMassSpectralAtlas...
17.27	988.77247	329.4	PE 34:1_20:4	$C_{59}H_{108}NO_8P$		SL		MSDIAL-TandemMassSpectralAtlas...

Analyte-list 200 -7 double -> 193								
RT [...	m/z meas.	CCS (...	Name	Molecular For...	Ions	Annotations	AQ	Annotation Source
14.13	716.52370	268.6	PE 16:0_18:1	$C_{39}H_{76}NO_8P$		AL		ClinLip Analyte List ...
13.56	714.50781	267.0	PE 16:0_18:2	$C_{39}H_{74}NO_8P$		AL		ClinLip Analyte List ...
13.21	738.50742	269.5	PE 16:0_20:4	$C_{41}H_{74}NO_8P$		AL		ClinLip Analyte List ...
13.52	762.50775	275.3	PE 16:0_22:6	$C_{43}H_{74}NO_8P$		AL		ClinLip Analyte List ...
12.79	674.47660	259.7	PE 17:0_14:1	$C_{36}H_{70}NO_8P$		AL		ClinLip Analyte List ...
14.96	744.55485	275.0	PE 18:0_18:1	$C_{41}H_{80}NO_8P$		AL		ClinLip Analyte List ...
14.45	742.53912	273.5	PE 18:0_18:2	$C_{41}H_{78}NO_8P$		AL		ClinLip Analyte List ...
14.48	766.53920	277.4	PE 18:0_20:4	$C_{43}H_{78}NO_8P$		AL		ClinLip Analyte List ...
14.41	790.53917	281.7	PE 18:0_22:6	$C_{45}H_{78}NO_8P$		AL		ClinLip Analyte List ...
13.83	740.52327	271.7	PE 18:1_18:2	$C_{41}H_{76}NO_8P$		AL		ClinLip Analyte List ...
13.83	764.52322	275.7	PE 18:1_20:4	$C_{43}H_{76}NO_8P$		AL		ClinLip Analyte List ...
13.59	738.50794	271.0	PE 18:2_18:2	$C_{41}H_{74}NO_8P$		AL		ClinLip Analyte List ...

Supplementary Table 2. Comparison of annotation sources. Comparison of lipid identification using two different annotation sources namely 1) MS Dial Spectral Library[3] and 2) in-house generated ClinLip Analyte List exemplified for phosphatidylethanolamine (PE) species. Red and yellow marks show miss identifications, green marks show annotations that are found by both annotation sources, and blue marks show PE species, only found via the ClinLip Analyte list.

Supplementary Table 3

(Set 1)

Lipid Class	ESTD	Conc. range (ng/mL)	ISTD		
			**Neg**	**Pos**	**Conc. (ng/mL)**
**Cer**	Cer d18:1_16:0	30, 60, 120, 240, 480, 960, 1920	Cer d18:1_17:0	Cer d18:1_17:0	75
**FAHFA**	Cer d18:1_16:0	30, 60, 120, 240, 480, 960, 1920	Cer d18:1_17:0		75
**SM**	SM d18:1_16:0	175, 350, 700, 1400, 2800, 5600, 11200	PC 17:0_14:1	SM d18:1_12:0 *	75
**LPC**	LPC 18:0	62.5, 125, 250, 500, 1000, 2000, 4000	LPC 17:1	LPC 17:1	75
**LPE**	LPE 18:0	10, 20, 40, 80, 160, 320, 640	LPE 17:1	LPE 17:1	75
**PC**	PC 16:0_18:1	156.25, 312.5, 625, 1250, 2500, 5000, 10000	PC 17:0_14:1	PC 17:0_14:1	75
**PE**	PE 16:0_18:1 (neg)<br>PE 18:0_18:1 (pos)	25, 50, 100, 200, 400, 800, 1600	PE 17:0_14:1	PC 17:0_14:1	75
**PI**	PI 16:0_18:1	50, 100, 200, 400, 800, 1600, 3200	PI 17:0_14:1	PI 17:0_14:1	75
**PG**	PG 16:0_18:1	2.5, 5, 10, 20, 40, 80, 160	PG 17:0_14:1	PG 17:0_14:1	75
**PS**	PS 16:0_18:1	37.5, 75, 150, 300, 600, 1200, 2400	PS 17:0_14:1	PS 17:0_14:1	75
**TG**	TG 16:1_16:1_18:1	125, 250, 500, 1000, 2000, 4000, 8000		TG 17:0_17:1_17:0 d5	75
**Chol**	Cholesterol	312.5, 625, 1250, 2500, 5000, 10000, 20000		Cholesterol d7	75
**DG**	PC 16:0_18:1	125, 250, 500, 1000, 2000, 4000, 8000		PC 17:0_14:1	75

* Endogenous traces detected in unspiked plasma extract were subtracted

Supplementary Table 3. List of Standards (Set 1). A full list of external (ESTD) and internal (ISTD) standards used for the quantification of each lipid class in both positive and negative modes along with their concentration(s) (ng/mL) (referred to as Set 1).

Supplementary Table 4

(Set 2)

Lipid Class	ESTD	Conc. range (ng/mL)	ISTD	Conc. (ng/mL)
**Cer**	Cer d18:1_18:1	30, 60, 120, 240, 480, 960, 1920	Cer d18:1_16:0 d7	300
**FAHFA**	FAHFA 16:0_18:0	18.75, 37.75, 75, 150, 300, 600, 1200	FAHFA 16:0_18:0 d9	150
**SM**	SM d18:1_16:0	50, 100, 200, 400, 800, 1600, 3200	SM 18:1_18:1 d9	300
**LPC**	LPC 18:0	37.5, 75, 150, 300, 600, 1200, 2400	LPC 17:0 d5	300
**LPE**	LPE 18:0	10, 20, 40, 80, 160, 320, 640	LPE 17:0 d5	150
**PC**	PC 16:0_18:1	100, 200, 400, 800, 1600, 3200, 6400	PC 17:0_18:1 d5	300
**PE**	PE 18:0_18:2	15, 30, 60, 120, 240, 480, 960	PE 17:0_14:1 d5	150
**PI**	PI 16:0_18:1	25, 50, 100, 200, 400, 800, 1600	PI 17:0_14:1 d5	150
**PG**	PG 16:0_18:1	2.5, 5, 10, 20, 40, 80, 160	PG 17:0_14:1 d5	50
**PS**	PS 16:0_18:1	18.75, 37.5, 75, 150, 300, 600, 1200	PS 17:0_14:1 d5	150
**Chol**	Cholesterol	312.5, 625, 1250, 2500, 5000, 10000, 20000	Cholesterol d7	300
**DG**	PC 16:0_18:1	100, 200, 400, 800, 1600, 3200, 6400	PC 17:0_18:1 d5	300
**TG**	TG 18:1_18:1_20:4	125, 250, 500, 1000, 2000, 4000, 8000	TG 14:0_16:1_14:0 d5	300

Supplementary Table 4. List of Standards (Set 2). A full list of standards that were used for the quantification of the inter-day extraction experiment, using deuterated internal standards (ISTDs) for every lipid class and adapted concentration(s) of external standards (ESTD) (ng/mL) (referred to as Set 2).

Supplementary Table 5

Name	RT [min]	CCS($Å^2$)	*m/z* meas.	plasma	plasma	plasma	serum	serum	serum
**PC 17:0_14:1**	12.72	280.2	718.53957	3910000	3169233	3369257	3853048	4182128	5258679
**PC 14:0_14:0**	11.91	274.7	678.50701	3969427	4206660	4978398	6976215	7089082	6194149
**PC 44:2**	17.71	310.5	898.72622	0	59853	0	0	392773	282853
**PC 44:4**	16.94	314.1	894.69363	538440	763202	570704	926182	1018111	901607

Supplementary Table 5. Endogenous internal standard (ISTD) intensities obtained via 4D lipidomics workflow. Represented are ISTDs reported in the literature for the quantification of the phosphatidylcholine (PC) lipid class and their endogenous response in NIST plasma and serum human standard reference material (SRM) obtained under the hereby used analytical conditions in positive ion mode.

Supplementary Table 6

			nmol/ml			Bowden
			PC 33:1 d7	PC 17:0_14:1	PC 17:0_14:1 - endogenous	
PC	PC 16:0_18:1	Manual	361.59	98.62	111.98	120
		Auto	422.45	133.28	160.50	
	PC 32:1	Manual	49.75	10.64	12.08	13
		Auto	52.73	12.90	15.50	
	PC 38:4	Manual	250.58	66.27	75.28	84
		Auto	269.48	80.78	97.20	

Supplementary Table 6. Internal standard (ISTD) performance in lipid extracts analyzed via 4D lipidomics workflow. Comparison of quantified phosphatidylcholine (PC) species, extracted via automated vs manual extraction protocol from NIST human plasma standard reference material (SRM) using different ISTDs and referenced to levels reported by Bowden et al.[4]. Source data are provided as a Source Data file.

Supplementary Table 7

			210625					210702					210723				
		Bowden	Plasma	Serum	Blood	DBS Venous	DBS finger	Plasma	Serum	Blood	DBS Venous	DBS finger	Plasma	Serum	Blood	DBS Venous	DBS finger
Cer d18:1_16:0	TOF - Neg	0.28	0.55	0.39	1.96	1.21	1.24	0.49	0.85	NQ	1.44	0.87	0.44	0.44	1.55	0.73	0.79
	TOF - Pos		0.33	0.49	2.10	1.29	1.17	0.42	0.52	NQ	1.17	0.87	0.43	0.53	1.76	0.82	0.85
	MRM		0.36	0.61	1.72	0.60	0.80	0.29	1.05	3.83	0.73	0.77	0.29	0.31	1.82	0.52	0.80
LPC 20:4 SN1	TOF - Neg	6	10.36	10.68	3.64	3.78	3.70	5.10	11.19	NQ	2.05	1.20	8.10	8.54	3.74	1.83	1.49
	TOF - Pos		11.46	9.63	4.69	3.47	2.99	6.40	13.84	NQ	4.00	1.53	8.90	9.05	4.48	2.15	1.94
	MRM		8.97	11.75	3.22	3.10	3.06	5.00	8.73	1.78	3.45	1.27	6.59	7.59	3.59	1.75	1.60
LPC 14:0 SN1	TOF - Neg	1	2.96	2.94	2.40	0.97	1.43	1.12	2.51	NQ	0.63	0.33	1.89	1.92	1.01	0.46	0.46
	TOF - Pos		2.59	2.31	3.01	1.58	1.49	1.34	2.52	NQ	0.99	0.40	1.82	2.02	1.31	0.58	0.51
	MRM		1.73	3.37	2.21	0.99	1.00	1.12	1.97	1.16	0.88	0.34	1.54	1.74	1.18	0.47	0.44
LPC 18:1 SN1	TOF - Neg	18	41.07	42.28	25.15	12.67	15.54	24.08	62.94	NQ	13.49	6.86	32.92	44.47	22.39	9.59	8.44
	TOF - Pos		30.67	24.31	28.52	9.08	18.85	22.38	54.87	NQ	17.77	6.82	28.10	32.66	22.05	10.21	9.14
	MRM		30.67	40.59	24.12	12.74	12.58	23.24	41.41	18.00	19.69	7.16	28.00	32.37	21.01	9.22	8.42
LPE 18:0 SN1	TOF - Neg	1.6	2.35	2.55	2.95	1.96	2.29	1.30	2.45	NQ	1.69	0.88	2.14	2.21	2.37	1.40	1.36
	TOF - Pos		2.44	3.21	2.92	1.77	2.33	1.24	2.05	NQ	1.90	0.86	2.01	2.58	2.12	1.36	1.18
	MRM		2.53	3.89	3.42	1.93	1.81	1.17	2.36	2.93	1.47	0.77	2.09	2.39	2.29	1.30	1.20
LPE 16:0 SN1	TOF - Neg	0.91	1.98	2.04	2.63	1.42	1.92	0.99	1.75	NQ	1.54	0.50	1.70	1.92	1.52	0.82	0.76
	TOF - Pos		1.88	3.53	2.45	1.37	1.46	1.00	1.81	NQ	1.26	0.57	2.01	2.39	1.59	0.93	0.87
	MRM		2.63	3.93	3.37	1.67	1.61	1.04	2.10	2.51	1.03	0.58	2.07	2.39	1.85	0.96	0.93
LPE 20:4 SN1	TOF - Neg	1.1	3.74	3.49	1.92	1.64	2.20	1.66	3.48	NQ	1.20	0.65	2.58	2.69	1.28	0.84	1.05
	TOF - Pos		3.38	3.21	1.59	1.66	1.67	1.67	3.16	NQ	1.55	0.69	2.68	2.75	1.25	0.88	0.98
	MRM		2.42	3.72	0.80	1.02	1.02	0.95	2.05	0.56	0.69	0.40	1.49	1.73	0.69	0.50	0.59
PC 34:1	TOF - Neg	120	419.05	363.92	536.94	202.13	231.66	294.04	498.96	NQ	247.48	144.83	319.39	345.05	296.81	154.74	168.25
	TOF - Pos		253.77	248.57	395.95	249.58	369.67	233.87	438.10	NQ	237.47	99.66	259.02	236.80	252.10	174.43	154.40
	MRM		337.11	401.11	359.02	167.13	211.06	246.79	413.28	537.61	200.36	115.89	232.62	297.84	290.08	139.99	136.96
PC 32:1	TOF - Neg	13	20.84	20.64	17.58	7.56	8.31	10.85	22.14	NQ	5.39	3.33	13.40	13.53	9.06	3.46	3.92
	TOF - Pos		24.94	24.77	31.47	12.84	17.95	13.48	29.76	NQ	13.84	4.64	17.25	20.70	18.21	6.41	6.09
	MRM		32.07	37.22	26.14	12.99	13.29	15.47	32.72	27.62	12.72	6.29	18.33	22.81	18.21	8.78	7.89
PC 38:4	TOF - Neg	84	159.49	158.55	60.29	56.09	57.84	88.69	168.55	NQ	54.96	32.68	111.14	128.15	84.86	40.02	30.95
	TOF - Pos		149.11	145.37	73.49	49.55	130.19	101.02	162.17	NQ	103.07	32.71	141.12	172.13	93.22	50.79	46.03
	MRM		103.12	129.29	37.48	30.42	31.86	69.57	131.33	54.10	32.87	16.59	68.59	87.91	49.39	22.29	21.18
PE 34:1	TOF - Neg	1.2	0.80	0.74	75.10	26.99	41.20	0.52	1.12	NQ	40.63	18.71	0.52	0.59	62.53	21.46	19.26
	TOF - Pos		0.99	1.89	43.22	21.46	27.86	0.77	1.48	NQ	29.62	12.20	0.81	1.13	38.91	16.14	13.84
	MRM		1.45	2.00	70.70	32.95	31.25	1.00	1.98	131.91	43.38	24.18	0.91	1.05	64.37	25.42	24.69
PE 38:6	TOF - Neg	3.2	1.79	1.80	5.11	5.02	7.77	1.01	1.92	NQ	8.02	3.40	2.35	2.29	7.82	4.00	3.56
	TOF - Pos		3.11	3.24	7.05	6.97	9.37	1.63	3.01	NQ	9.79	4.13	3.44	3.91	11.40	5.55	5.33
	MRM																
PE 38:4	TOF - Neg	8.1	7.31	7.37	20.80	20.20	30.82	4.15	7.75	NQ	28.55	13.73	5.59	6.18	32.59	14.16	14.30
	TOF - Pos		7.48	6.91	21.67	20.54	27.14	4.47	8.02	NQ	26.05	11.07	6.46	8.77	32.17	13.66	12.88
	MRM		3.92	6.03	9.22	8.91	8.37	2.29	4.46	20.14	11.10	6.38	2.80	3.47	15.42	6.30	6.40
PE 40:6	TOF - Neg	1.8	1.33	1.44	1.66	1.62	2.49	0.72	1.33	NQ	2.16	1.02	1.56	1.80	3.00	1.44	1.32
	TOF - Pos		2.24	2.05	2.64	2.85	2.85	1.14	2.19	NQ	3.39	1.40	2.53	3.87	4.72	1.98	1.92
	MRM																
PG 34:1	TOF - Neg		0.12	0.03	0.29	0.19	0.10	0.04	0.13	NQ	0.20	0.13	0.13	0.09	0.33	0.10	0.10
	TOF - Pos																
	MRM		0.13	0.18	0.30	0.19	0.12	0.05	0.11	0.35	0.22	0.11	0.10	0.13	0.31	0.15	0.13
PI 34:1	TOF - Neg	2.4	1.49	1.41	1.54	0.80	1.16	2.75	4.11	NQ	1.51	0.89	1.53	1.77	1.07	0.62	0.56
	TOF - Pos		3.41	9.94	2.02	1.31	4.35	2.72	9.78	NQ	2.14	1.48	2.42	3.13	0.61	1.07	1.29
	MRM		2.64	3.92	2.78	1.30	1.50	3.03	6.52	3.70	2.58	1.35	2.22	2.45	2.45	0.97	1.08
PI 36:4	TOF - Neg	3	2.34	2.23	1.64	0.61	1.58	1.67	2.93	NQ	1.52	0.88	1.92	2.21	2.06	0.99	0.56
	TOF - Pos		3.82	7.77	1.62	2.38	1.96	2.01	5.62	NQ	2.42	1.41	2.72	3.58	1.67	1.30	1.39
	MRM		3.11	4.90	1.83	1.66	1.52	1.54	3.79	2.25	1.84	1.07	2.07	2.54	2.35	0.99	0.99
PI 38:4	TOF - Neg	19	30.16	27.81	20.77	13.39	18.24	25.50	45.66	NQ	20.69	11.25	32.10	37.87	29.64	11.95	6.81
	TOF - Pos																
	MRM		23.53	36.09	13.21	11.07	10.34	14.85	35.66	21.16	14.93	8.30	19.62	23.42	20.34	8.41	7.80
SM 36:1	TOF - Neg	20	12.73	11.77	24.12	13.81	15.94	13.34	19.36	NQ	15.68	11.09	11.51	14.07	16.04	8.90	8.54
	TOF - Pos		28.88	27.61	29.53	14.46	17.69	24.94	28.43	NQ	22.53	13.66	23.80	26.58	24.38	16.75	18.49
	MRM		20.00	22.96	23.04	11.99	12.14	17.69	26.33	37.12	15.50	11.55	17.80	18.84	23.49	11.36	10.94
SM 42:1	TOF - Neg	20	19.22	18.66	70.21	53.72	57.00	20.02	27.89	NQ	66.07	46.33	15.15	13.68	44.18	35.94	20.39
	TOF - Pos		15.82	17.00	46.08	18.87	22.18	12.30	18.64	NQ	28.94	19.67	12.16	18.40	42.79	21.13	22.73
	MRM		8.61	9.86	34.63	15.77	15.35	7.47	11.69	99.50	19.41	13.52	6.88	7.32	31.00	13.33	12.59
		Bowden	Plasma	Serum	Blood	DBS Venous	DBS finger	Plasma	Serum	Blood	DBS Venous	DBS finger	Plasma	Serum	Blood	DBS Venous	DBS finger

Supplementary Table 7. Quantified values of lipid species from the LBlooD study. The table represents the comparison of the quantified values (nmol/mL) of 20 lipid species identified in both positive and negative mode by trapped ion mobility-Time of Flight (timsTOF) and by multiple reaction monitoring (MRM) analysis (with negative and positive ion mode switching) for all biological matrices of an individual over three-time points. Source data are provided in Supplementary Data 9 and 10.

Supplementary Table 8

(Quality control (QC))

Lipid Class	ESTD	ISTD
**Cer**	Cer d18:1_16:0	Cer d18:1_17:0
**SM**	SM d18:1_16:0	SM d18:1_12:0
**LPC**	LPC 18:0	LPC 17:1
**PC**	PC 18:0_14:0<br>PC 14:0_18:0<br>PC 16:0_16:0<br>PC 24:0_24:0	PC 17:0_14:1
**LPA**	LPA 18:0	LPA 17:0
**PA**	PA 16:0_18:1<br>PA 16:0_20:4	PA 15:0_18:1 d7
**LPE**	LPE 18:0	LPE 17:1
**PE**	PE 16:0_18:1<br>PE 18:0_18:0	PE 17:0_14:1
**LPI**	LPI 18:0	LPI 17:1
**PI**	PI 16:0_18:1	PI 17:0_14:1
**LPS**	LPS 18:1	LPS 17:1
**PS**	PS 16:0_18:1<br>PS 18:0_18:2	PS 17:0_14:1
**LPG**	LPG 18:0	LPG 17:1
**PG**	PG 18:1_18:1 Cis<br>PG 18:1_18:1 Trans	PG 17:0_14:1
**SP**	S1P 18:1	S1P 17:1
**CP**	CerP(d18:1/16:0)	CerP (d18:1/12:0)

Supplementary Table 8. List of standards (QC). A full list of external (ESTD) and internal (ISTD) standards used for quality control analysis.

Supplementary References

1. Zhou, Z., Tu, J., Xiong, X., Shen, X. & Zhu, Z. J. LipidCCS: Prediction of Collision Cross-Section Values for Lipids with High Precision to Support Ion Mobility-Mass Spectrometry-Based Lipidomics. *Anal Chem* **89**, 9559–9566 (2017).

2. Vasilopoulou, C. G. *et al.* Trapped ion mobility spectrometry and PASEF enable in-depth lipidomics from minimal sample amounts. *Nat Commun* **11**, 1–11 (2020).

3. Tsugawa, H. *et al.* A lipidome atlas in MS-DIAL 4. *Nat Biotechnol* **38**, 1159–1163 (2020).

4. Bowden, J. A., Ulmer, C. Z., Jones, C. M. & Heckert, A. Lipid Concentrations in Standard Reference Material (SRM) 1950 : Results from an Interlaboratory Comparison Exercise for Lipidomics Lipid Concentrations in Standard Reference Material (SRM) 1950 : Results from an Interlaboratory Comparison Exercise fo. *Nistir* **8185**, 1–451 (2017).

5. Nelson, A. B., Chow, L. S., Hughey, C. C., Crawford, P. A. & Puchalska, P. Artifactual FA dimers mimic FAHFA signals in untargeted metabolomics pipelines. *J Lipid Res* **64**, 100201 (2022).

6. Wolrab, D., Chocholoušková, M., Jirásko, R., Peterka, O. & Holčapek, M. Validation of lipidomic analysis of human plasma and serum by supercritical fluid chromatography–mass spectrometry and hydrophilic interaction liquid chromatography–mass spectrometry. *Anal Bioanal Chem* **412**, 2375–2388 (2020).
