# Supplementary material for: Four-dimensional trapped ion mobility spectrometry lipidomics for high throughput clinical profiling of human blood samples
Source: Nat Commun. 2023 Feb 20;14:937. doi: 10.1038/s41467-023-36520-1 (PMC9941096; doi:10.1038/s41467-023-36520-1)
Supplement: Supplementary file 3 — Description of Additional Supplementary Files [file 41467_2023_36520_MOESM3_ESM.pdf]

Description of Additional Supplementary Files

File Name: Supplementary Data 1
Description: **Clinical lipidomics analyte list from calibration standard**. Full list of all the lipid standards used for the generation of the in-house library in both positive (200) and negative mode (183) with their respective retention time (RT), collisional cross section (CCS), and neutral mass in negative and positive ion modes.

File Name: Supplementary Data 2
Description: **Collisional cross section** (**CCS) based curation**. List of lipid species for which multiple features were obtained and for which CCS descriptor was used to identify the correct feature/annotation for that molecule.

File Name: Supplementary Data 3
Description: **Clinical lipidomics analyte list from calibration standards and plasma**. Analyte list in both positive (424) and negative mode (391) containing retention time (RT), collisional cross section (CCS), neutral mass, and MS2 information of the lipid standards (screened individually), as well as the lipid species manually curated from NIST human plasma standard reference material (SRM).

File Name: Supplementary Data 4
Description: **Annotated and quantified values from NIST human serum standard reference material (SRM)**. Table representing the annotated and quantified values (nmol/mL) from NIST serum SRM of all the lipid species identified in negative (176) and positive (302) mode using the multi-point strategy along with their molecular descriptors.

File Name: Supplementary Data 5
Description: **Stable unknown features**. Table displaying the raw data for assessment of the stable unknown features. Features from the repetition experiment were analyzed for overlapping with the plasma dilution experiment using the Pearson correlation. Identified overlapping features were accepted under two criteria: (1) the value of the feature was positively correlated with the dilution using the Pearson correlation ($p \geq 0.9$) and (2) the SD from the mean of the dilution experiments was > 0.1. Accepted features are color-coded depending on their correlation value as follows: green, $p \geq 0.9$; yellow, $0.75 \geq p > 0.9$. Gray marks features overlapping with the features from the dilution experiment but not exhibiting a dilution response.

File Name: Supplementary Data 6
Description: **Quantified values of plasma lipids and their descriptors from the intra-day extraction analysis using set 1 standards.** Table representing the quantified values (nmol/mL) from NIST human plasma standard reference material (SRM) of all the lipid species identified in negative (180) and positive (315) mode using the multi-point strategy compared to quantified values reported by Bowden et. al.[4] and Wolrab et. al. [6]. The list also comprises of the molecular descriptors (retention time (RT), collisional cross section (CCS), *m/z*, 4D peak area, MS2 fragment annotations), coefficient of variation for RT, CCS and average of the mass error (in ppm) for 64 measurements (measurement of 32 extracts and remeasurement thereof) in negative and 32 measurements in positive mode. The table also contains a comparison of the quantified values (nmol/mL) of all the common lipid species identified in both positive and negative mode (125) using the multi-point strategy with quantified values reported by Bowden et. al.[4] and Wolrab et. al.[6] Table also lists a comparison of the quantified lipid species in positive and negative mode from the tims-TOF measurements with quantified values from MRM analysis.

File Name: Supplementary Data 7
Description: **Quantified values of plasma lipids from the inter-day extraction analysis using a different set of standards (set 2 standards)**. The table shows the comparison of quantified values of plasma lipids between intra-day measurements quantified using set 1 standards (n = 32) and inter-day extraction performed with set 2 (containing only deuterated ISTDs) standards. The inter-day extraction was performed on two different days (n = 32 per day and plate) respectively, one year after the first intra-day extraction (set 1, Supplementary Data 6), as well as the coefficient of variation across the measurements for both studies. The list also comprises the molecular descriptors (retention time (RT), collisional cross section (CCS), *m/z*, 4D peak area) for all the lipid species observed for inter-day extraction in both polarities.

File Name: Supplementary Data 8
Description: **CERT2 Score for NIST human plasma standard reference material (SRM) and participating individuals**. The figure shows the consistency of the calculated CERT2 Score for two individuals in the LBlooD study and NIST plasma SRM data from intra-day and inter-day extraction assays.

File Name: Supplementary Data 9
Description: **Quantified values from the LBlooD study (negative mode)**. Full list of quantified values of all the lipid species identified by negative mode analysis in the five biological matrices (plasma, serum, blood, dried blood spots (DBS) Venous, and DBS Finger) over the three-time points for all the participating individuals in the experiment.

File Name: Supplementary Data 10
Description: **Quantified values from the LBlooD study (positive mode)**. Full list of quantified values of all the lipid species identified by positive mode analysis in the five biological matrices (plasma, serum, blood, dried blood spots (DBS) Venous, and DBS Finger) over the three-time points for all the participating individuals in the experiment.

File Name: Supplementary Data 11
Description: **epMotion® script- extraction**. epMotion® scripts for lipid extraction.

File Name: Supplementary Data 12
Description: **epMotion® script- resolving**. epMotion® scripts for lipid-resolving.

File Name: Supplementary Data 13
Description: **Mass and mobility recalibration**. List of Peaks from the tune-mix and sodium formate mixture used for the recalibration of mass and mobility values in the raw data.

File Name: Supplementary Data 14
Description: **Regression parameters for the quantification curves.** The table lists the regression coefficients used for the multi-point quantification strategy in both polarities for inter-day measurement (set 1 standards) and inter-day extraction (set 2 standards) analysis. The list also contains the coefficient of distribution (R2) used to determine the linearity of quantification curves. For most of the quantification curves, R2 ≥ 0.99 was observed.

File Name: Supplementary Data 15
Description: **Python script for unknown feature correlation.** The data file shows the python script used for the correlation of the unknown features with the NIST human plasma standard reference material (SRM) dilution experiment; used for Supplementary Data 5.
