# Supplementary material for: Four-dimensional trapped ion mobility spectrometry lipidomics for high throughput clinical profiling of human blood samples
Source: Nat Commun. 2023 Feb 20;14:937. doi: 10.1038/s41467-023-36520-1 (PMC9941096; doi:10.1038/s41467-023-36520-1)
Supplement: Supplementary file 14 — Supplementary Data 11 [file 41467_2023_36520_MOESM14_ESM.pdf]

Eppendorf AG
Barkhausenweg 1
22339 Hamburg
Germany

9/13/2022

**User**	user
**Device**	5073JK706157
**Logfile**	
**Logfile Path**	Home
**Logfile Created**	
**Software version**	Device: 18.4 Server: 40.7.2.8
**Application**	
**Created**	
**Last modified**	
**Path**	Home
**Comment**	

Eppendorf epMotion

- -

Method name and path :

Date of Run :
Last edit :
Firmware version : epMotion 18.04
Software version : 40.7.2.8
TIME----- COMMAND------------ INFORMATION------------------------------
4:19:08 PM Program Init
4:19:08 PM Program Collect
4:19:12 PM !Dialog Text: Number of samples
Reply: 1
Answer: 32
4:19:15 PM Program Check
4:19:15 PM Application runner settings
Levels: ON
Tips: ON
Locations: ON

Vessel caps: OFF
Auto tool selection: OFF
4:19:26 PM HEPA unit: OFF
4:19:26 PM !!! WARNING !!! Device setup is invalid. Please contact service.
4:19:37 PM Location scan: Slot TMX, height 45.913 mm
4:19:36 PM Level Name: DWP 2mL, Labware: dws/plates/dwp96/EP_DWP_2000_1
Position: A1, Volume: 0µl
Position: A2, Volume: 0µl
Position: A3, Volume: 0µl
Position: A4, Volume: 0µl
Position: B1, Volume: 0µl
Position: B2, Volume: 0µl
Position: B3, Volume: 0µl
Position: B4, Volume: 0µl
Position: C1, Volume: 0µl
Position: C2, Volume: 0µl
Position: C3, Volume: 0µl
Position: C4, Volume: 0µl
Position: D1, Volume: 0µl
Position: D2, Volume: 0µl
Position: D3, Volume: 0µl
Position: D4, Volume: 0µl
Position: E1, Volume: 0µl
Position: E2, Volume: 0µl
Position: E3, Volume: 0µl
Position: E4, Volume: 0µl
Position: F1, Volume: 0µl
Position: F2, Volume: 0µl
Position: F3, Volume: 0µl
Position: F4, Volume: 0µl
Position: G1, Volume: 0µl
Position: G2, Volume: 0µl
Position: G3, Volume: 0µl
Position: G4, Volume: 0µl
Position: H1, Volume: 0µl
Position: H2, Volume: 0µl
Position: H3, Volume: 0µl
Position: H4, Volume: 0µl
4:19:41 PM Location scan: Slot A2, height 99.767 mm
4:19:55 PM Genuine Eppendorf tips found in slot A2

Eppendorf AG
Barkhausenweg 1
22339 Hamburg
Germany

4:20:07 PM Tip Scan Name: tip1000f_1, Labware: dws/tips/tip1000f, Number of tips: 16
4:20:11 PM Location scan: Slot B1, height 43.022 mm
4:20:11 PM Level Name: DWP_1mL, Labware: dws/plates/dwp96/EP_DWP_1000_1
Position: A1, Volume: 0µl
Position: A2, Volume: 0µl
Position: A3, Volume: 0µl
Position: A4, Volume: 0µl
Position: B1, Volume: 0µl
Position: B2, Volume: 0µl
Position: B3, Volume: 0µl
Position: B4, Volume: 0µl
Position: C1, Volume: 0µl
Position: C2, Volume: 0µl
Position: C3, Volume: 0µl
Position: C4, Volume: 0µl
Position: D1, Volume: 0µl
Position: D2, Volume: 0µl
Position: D3, Volume: 0µl
Position: D4, Volume: 0µl
Position: E1, Volume: 0µl
Position: E2, Volume: 0µl
Position: E3, Volume: 0µl
Position: E4, Volume: 0µl
Position: F1, Volume: 0µl
Position: F2, Volume: 0µl
Position: F3, Volume: 0µl
Position: F4, Volume: 0µl
Position: G1, Volume: 0µl
Position: G2, Volume: 0µl
Position: G3, Volume: 0µl
Position: G4, Volume: 0µl
Position: H1, Volume: 0µl
Position: H2, Volume: 0µl
Position: H3, Volume: 0µl
Position: H4, Volume: 0µl
4:20:15 PM Location scan: Slot B2, height 58.974 mm
4:20:30 PM Genuine Eppendorf tips found in slot B2
4:20:43 PM Tip Scan Name: tip300f_1, Labware: dws/tips/tip300f, Number of tips: 24
4:21:39 PM !Dialog Text: Checking
Reply: 1

Eppendorf AG
Barkhausenweg 1
22339 Hamburg
Germany

Answer: Repeat scan
4:21:58 PM !Dialog Text: Checking
Reply: 1
Answer: Repeat scan
4:22:53 PM Level Name: SOLVENTS, Labware: dws/th/solvents
Position: 1, Volume: 59827.5µl
Position: 2, Volume: 15982µl
Position: 3, Volume: 7694µl
4:22:58 PM Location scan: Slot C1, height 59.010 mm
4:23:13 PM Genuine Eppendorf tips found in slot C1
4:23:25 PM Tip Scan Name: tip50f_1, Labware: dws/tips/tip50f, Number of tips: 72
4:23:28 PM Tool Scan Position: Gripper, Code: N/A
4:23:31 PM Tool Scan Position: T1, Code: D_50_8, serial number: 1002123
4:23:34 PM Tool Scan Position: T2, Code: D_1000_8, serial number: 421352I
4:23:37 PM Tool Scan Position: T3, Code: D_300_8, serial number: 224322K
4:23:50 PM 1 Thermomixer Temperature: 8°C
Current Temperature: 20°C
4:23:50 PM 2 Comment Transfer Probe in 2mL DWP
4:23:50 PM 3 Comment Zugabe MTBE
4:23:50 PM 4 Reagent Transfer
Source:
Name: SOLVENTS, Labware: dws/th/solvents
Destination:
Name: DWP 2mL, Labware: dws/plates/dwp96/EP_DWP_2000_1
Number of samples: 32
Tool: TM_1000_8, Filter tips: Yes, Liquid: Alcohol 98%
Volume: 615µl, Transfer type: Pipette
Change tips: when command finished after 6 aspirations
Dispose tips into waste.
Options: Aspirate from bottom, dispense 5 from liquid level, follow liquid level change
Changes in liquid types:
Speed aspiration: 4.0mm/sec, Speed dispense: 4.0mm/sec
Liquid offset aspiration: 3.0mm, Liquid offset dispense: -3.0mm
Delay blow: 0ms, Speed blow: 0.4mm/sec
Movement blow: 0%, Initial stroke: 100%
Prewetting: 1 cycles
4:24:02 PM New tip acquired from tip1000f_1, A11
4:24:32 PM Source: SOLVENTS, Position: 1, Well volume: 54907.5µl
4:24:40 PM Destination: DWP 2mL, Position: A1, Well volume: 615.0µl
4:24:40 PM Destination: DWP 2mL, Position: B1, Well volume: 615.0µl

Eppendorf AG
Barkhausenweg 1
22339 Hamburg
Germany

4:24:40 PM Destination: DWP 2mL, Position: C1, Well volume: 615.0µl
4:24:40 PM Destination: DWP 2mL, Position: D1, Well volume: 615.0µl
4:24:40 PM Destination: DWP 2mL, Position: E1, Well volume: 615.0µl
4:24:40 PM Destination: DWP 2mL, Position: F1, Well volume: 615.0µl
4:24:40 PM Destination: DWP 2mL, Position: G1, Well volume: 615.0µl
4:24:40 PM Destination: DWP 2mL, Position: H1, Well volume: 615.0µl
4:24:53 PM Source: SOLVENTS, Position: 1, Well volume: 49987.5µl
4:25:01 PM Destination: DWP 2mL, Position: A2, Well volume: 615.0µl
4:25:01 PM Destination: DWP 2mL, Position: B2, Well volume: 615.0µl
4:25:01 PM Destination: DWP 2mL, Position: C2, Well volume: 615.0µl
4:25:01 PM Destination: DWP 2mL, Position: D2, Well volume: 615.0µl
4:25:01 PM Destination: DWP 2mL, Position: E2, Well volume: 615.0µl
4:25:01 PM Destination: DWP 2mL, Position: F2, Well volume: 615.0µl
4:25:01 PM Destination: DWP 2mL, Position: G2, Well volume: 615.0µl
4:25:01 PM Destination: DWP 2mL, Position: H2, Well volume: 615.0µl
4:25:15 PM Source: SOLVENTS, Position: 1, Well volume: 45067.5µl
4:25:22 PM Destination: DWP 2mL, Position: A3, Well volume: 615.0µl
4:25:22 PM Destination: DWP 2mL, Position: B3, Well volume: 615.0µl
4:25:22 PM Destination: DWP 2mL, Position: C3, Well volume: 615.0µl
4:25:22 PM Destination: DWP 2mL, Position: D3, Well volume: 615.0µl
4:25:22 PM Destination: DWP 2mL, Position: E3, Well volume: 615.0µl
4:25:22 PM Destination: DWP 2mL, Position: F3, Well volume: 615.0µl
4:25:22 PM Destination: DWP 2mL, Position: G3, Well volume: 615.0µl
4:25:22 PM Destination: DWP 2mL, Position: H3, Well volume: 615.0µl
4:25:36 PM Source: SOLVENTS, Position: 1, Well volume: 40147.5µl
4:25:43 PM Destination: DWP 2mL, Position: A4, Well volume: 615.0µl
4:25:43 PM Destination: DWP 2mL, Position: B4, Well volume: 615.0µl
4:25:43 PM Destination: DWP 2mL, Position: C4, Well volume: 615.0µl
4:25:43 PM Destination: DWP 2mL, Position: D4, Well volume: 615.0µl
4:25:43 PM Destination: DWP 2mL, Position: E4, Well volume: 615.0µl
4:25:43 PM Destination: DWP 2mL, Position: F4, Well volume: 615.0µl
4:25:43 PM Destination: DWP 2mL, Position: G4, Well volume: 615.0µl
4:25:43 PM Destination: DWP 2mL, Position: H4, Well volume: 615.0µl
4:25:50 PM Tip disposed.
4:25:54 PM 5 Comment Zugabe 200µL 0,1% FA
4:25:54 PM 6 Reagent Transfer
Source:
Name: SOLVENTS, Labware: dws/th/solvents
Destination:
Name: DWP 2mL, Labware: dws/plates/dwp96/EP_DWP_2000_1

Eppendorf AG
Barkhausenweg 1
22339 Hamburg
Germany

Number of samples: 32
Tool: TM_300_8, Filter tips: Yes, Liquid: Water
Volume: 200µl, Transfer type: Pipette
Change tips: when command finished after 6 aspirations
Dispose tips into waste.
Options: dispense 5 from liquid level, follow liquid level change
4:26:17 PM New tip acquired from tip300f_1, A10
4:26:22 PM Source: SOLVENTS, Position: 2, Well volume: 14382.0µl
4:26:26 PM Destination: DWP 2mL, Position: A1, Well volume: 815.0µl
4:26:26 PM Destination: DWP 2mL, Position: B1, Well volume: 815.0µl
4:26:26 PM Destination: DWP 2mL, Position: C1, Well volume: 815.0µl
4:26:26 PM Destination: DWP 2mL, Position: D1, Well volume: 815.0µl
4:26:26 PM Destination: DWP 2mL, Position: E1, Well volume: 815.0µl
4:26:26 PM Destination: DWP 2mL, Position: F1, Well volume: 815.0µl
4:26:26 PM Destination: DWP 2mL, Position: G1, Well volume: 815.0µl
4:26:26 PM Destination: DWP 2mL, Position: H1, Well volume: 815.0µl
4:26:30 PM Source: SOLVENTS, Position: 2, Well volume: 12782.0µl
4:26:34 PM Destination: DWP 2mL, Position: A2, Well volume: 815.0µl
4:26:34 PM Destination: DWP 2mL, Position: B2, Well volume: 815.0µl
4:26:34 PM Destination: DWP 2mL, Position: C2, Well volume: 815.0µl
4:26:34 PM Destination: DWP 2mL, Position: D2, Well volume: 815.0µl
4:26:34 PM Destination: DWP 2mL, Position: E2, Well volume: 815.0µl
4:26:34 PM Destination: DWP 2mL, Position: F2, Well volume: 815.0µl
4:26:34 PM Destination: DWP 2mL, Position: G2, Well volume: 815.0µl
4:26:34 PM Destination: DWP 2mL, Position: H2, Well volume: 815.0µl
4:26:39 PM Source: SOLVENTS, Position: 2, Well volume: 11182.0µl
4:26:42 PM Destination: DWP 2mL, Position: A3, Well volume: 815.0µl
4:26:42 PM Destination: DWP 2mL, Position: B3, Well volume: 815.0µl
4:26:42 PM Destination: DWP 2mL, Position: C3, Well volume: 815.0µl
4:26:42 PM Destination: DWP 2mL, Position: D3, Well volume: 815.0µl
4:26:42 PM Destination: DWP 2mL, Position: E3, Well volume: 815.0µl
4:26:42 PM Destination: DWP 2mL, Position: F3, Well volume: 815.0µl
4:26:42 PM Destination: DWP 2mL, Position: G3, Well volume: 815.0µl
4:26:42 PM Destination: DWP 2mL, Position: H3, Well volume: 815.0µl
4:26:47 PM Source: SOLVENTS, Position: 2, Well volume: 9582.0µl
4:26:50 PM Destination: DWP 2mL, Position: A4, Well volume: 815.0µl
4:26:50 PM Destination: DWP 2mL, Position: B4, Well volume: 815.0µl
4:26:50 PM Destination: DWP 2mL, Position: C4, Well volume: 815.0µl
4:26:50 PM Destination: DWP 2mL, Position: D4, Well volume: 815.0µl
4:26:50 PM Destination: DWP 2mL, Position: E4, Well volume: 815.0µl

4:26:50 PM Destination: DWP 2mL, Position: F4, Well volume: 815.0µl
4:26:50 PM Destination: DWP 2mL, Position: G4, Well volume: 815.0µl
4:26:50 PM Destination: DWP 2mL, Position: H4, Well volume: 815.0µl
4:26:58 PM Tip disposed.
4:27:02 PM 7 Comment Spike ISTD
4:27:02 PM 8 Reagent Transfer
Source:
Name: SOLVENTS, Labware: dws/th/solvents
Destination:
Name: DWP 2mL, Labware: dws/plates/dwp96/EP_DWP_2000_1
Number of samples: 32
Tool: TM_300_8, Filter tips: Yes, Liquid: Alcohol 98%
Volume: 185µl, Transfer type: Pipette
Change tips: before each aspiration
Dispose tips into waste.
Options: dispense 5 from liquid level, follow liquid level change
4:27:07 PM New tip acquired from tip300f_1, A11
4:27:20 PM Source: SOLVENTS, Position: 3, Well volume: 6214.0µl
4:27:25 PM Destination: DWP 2mL, Position: A1, Well volume: 1000.0µl
4:27:25 PM Destination: DWP 2mL, Position: B1, Well volume: 1000.0µl
4:27:25 PM Destination: DWP 2mL, Position: C1, Well volume: 1000.0µl
4:27:25 PM Destination: DWP 2mL, Position: D1, Well volume: 1000.0µl
4:27:25 PM Destination: DWP 2mL, Position: E1, Well volume: 1000.0µl
4:27:25 PM Destination: DWP 2mL, Position: F1, Well volume: 1000.0µl
4:27:25 PM Destination: DWP 2mL, Position: G1, Well volume: 1000.0µl
4:27:25 PM Destination: DWP 2mL, Position: H1, Well volume: 1000.0µl
4:27:32 PM Tip disposed.
4:27:40 PM New tip acquired from tip300f_1, A12
4:27:53 PM Source: SOLVENTS, Position: 3, Well volume: 4734.0µl
4:27:58 PM Destination: DWP 2mL, Position: A2, Well volume: 1000.0µl
4:27:58 PM Destination: DWP 2mL, Position: B2, Well volume: 1000.0µl
4:27:58 PM Destination: DWP 2mL, Position: C2, Well volume: 1000.0µl
4:27:58 PM Destination: DWP 2mL, Position: D2, Well volume: 1000.0µl
4:27:58 PM Destination: DWP 2mL, Position: E2, Well volume: 1000.0µl
4:27:58 PM Destination: DWP 2mL, Position: F2, Well volume: 1000.0µl
4:27:58 PM Destination: DWP 2mL, Position: G2, Well volume: 1000.0µl
4:27:58 PM Destination: DWP 2mL, Position: H2, Well volume: 1000.0µl
4:28:05 PM Tip disposed.
4:28:18 PM !Dialog Text: Please insert new tips for 300 µl with filter in slot B2 (tip300f_1)

WARNING: Please also make sure that you remove all restored tips of the given type.
Reply: 1
4:28:30 PM Location scan: Slot B2, height 58.819 mm
4:28:45 PM Genuine Eppendorf tips found in slot B2
4:28:58 PM Tip Scan Name: tip300f_1, Labware: dws/tips/tip300f, Number of tips: 32
4:29:11 PM New tip acquired from tip300f_1, A9
4:29:24 PM Source: SOLVENTS, Position: 3, Well volume: 3254.0µl
4:29:29 PM Destination: DWP 2mL, Position: A3, Well volume: 1000.0µl
4:29:29 PM Destination: DWP 2mL, Position: B3, Well volume: 1000.0µl
4:29:29 PM Destination: DWP 2mL, Position: C3, Well volume: 1000.0µl
4:29:29 PM Destination: DWP 2mL, Position: D3, Well volume: 1000.0µl
4:29:29 PM Destination: DWP 2mL, Position: E3, Well volume: 1000.0µl
4:29:29 PM Destination: DWP 2mL, Position: F3, Well volume: 1000.0µl
4:29:29 PM Destination: DWP 2mL, Position: G3, Well volume: 1000.0µl
4:29:29 PM Destination: DWP 2mL, Position: H3, Well volume: 1000.0µl
4:29:36 PM Tip disposed.
4:29:44 PM New tip acquired from tip300f_1, A10
4:29:57 PM Source: SOLVENTS, Position: 3, Well volume: 1774.0µl
4:30:02 PM Destination: DWP 2mL, Position: A4, Well volume: 1000.0µl
4:30:02 PM Destination: DWP 2mL, Position: B4, Well volume: 1000.0µl
4:30:02 PM Destination: DWP 2mL, Position: C4, Well volume: 1000.0µl
4:30:02 PM Destination: DWP 2mL, Position: D4, Well volume: 1000.0µl
4:30:02 PM Destination: DWP 2mL, Position: E4, Well volume: 1000.0µl
4:30:02 PM Destination: DWP 2mL, Position: F4, Well volume: 1000.0µl
4:30:02 PM Destination: DWP 2mL, Position: G4, Well volume: 1000.0µl
4:30:02 PM Destination: DWP 2mL, Position: H4, Well volume: 1000.0µl
4:30:10 PM Tip disposed.
4:30:14 PM 9 User Intervention Comment: Cover 2mL DWP with mat
after that the epMotion will vortex for 10min at 1050 rpm
Alarm: On
4:31:14 PM continue
4:31:14 PM 10 Thermomixer Speed: 1050rpm, Time: 10:00
Temperature: 8°C
Current Temperature: 11°C
4:31:14 PM 11 User InterventionComment: centrifugate 2mL DWP for 5min, 4 C & 5.000 g
afterwards replace in the epMotion
Alarm: On
4:54:42 PM continue
4:54:40 PM12 Set volume (absolute)

Labware: DWP 2mL
Well A1: changed to 1600 µl
Well A2: changed to 1600 µl
Well A3: changed to 1600 µl
Well A4: changed to 1600 µl
Well A5: changed to 1600 µl
Well A6: changed to 1600 µl
Well A7: changed to 1600 µl
Well A8: changed to 1600 µl
Well A9: changed to 1600 µl
Well A10: changed to 1600 µl
Well A11: changed to 1600 µl
Well A12: changed to 1600 µl
Well B1: changed to 1600 µl
Well B2: changed to 1600 µl
Well B3: changed to 1600 µl
Well B4: changed to 1600 µl
Well B5: changed to 1600 µl
Well B6: changed to 1600 µl
Well B7: changed to 1600 µl
Well B8: changed to 1600 µl
Well B9: changed to 1600 µl
Well B10: changed to 1600 µl
Well B11: changed to 1600 µl
Well B12: changed to 1600 µl
Well C1: changed to 1600 µl
Well C2: changed to 1600 µl
Well C3: changed to 1600 µl
Well C4: changed to 1600 µl
Well C5: changed to 1600 µl
Well C6: changed to 1600 µl
Well C7: changed to 1600 µl
Well C8: changed to 1600 µl
Well C9: changed to 1600 µl
Well C10: changed to 1600 µl
Well C11: changed to 1600 µl
Well C12: changed to 1600 µl
Well D1: changed to 1600 µl
Well D2: changed to 1600 µl
Well D3: changed to 1600 µl

Well D4: changed to 1600 µl
Well D5: changed to 1600 µl
Well D6: changed to 1600 µl
Well D7: changed to 1600 µl
Well D8: changed to 1600 µl
Well D9: changed to 1600 µl
Well D10: changed to 1600 µl
Well D11: changed to 1600 µl
Well D12: changed to 1600 µl
Well E1: changed to 1600 µl
Well E2: changed to 1600 µl
Well E3: changed to 1600 µl
Well E4: changed to 1600 µl
Well E5: changed to 1600 µl
Well E6: changed to 1600 µl
Well E7: changed to 1600 µl
Well E8: changed to 1600 µl
Well E9: changed to 1600 µl
Well E10: changed to 1600 µl
Well E11: changed to 1600 µl
Well E12: changed to 1600 µl
Well F1: changed to 1600 µl
Well F2: changed to 1600 µl
Well F3: changed to 1600 µl
Well F4: changed to 1600 µl
Well F5: changed to 1600 µl
Well F6: changed to 1600 µl
Well F7: changed to 1600 µl
Well F8: changed to 1600 µl
Well F9: changed to 1600 µl
Well F10: changed to 1600 µl
Well F11: changed to 1600 µl
Well F12: changed to 1600 µl
Well G1: changed to 1600 µl
Well G2: changed to 1600 µl
Well G3: changed to 1600 µl
Well G4: changed to 1600 µl
Well G5: changed to 1600 µl
Well G6: changed to 1600 µl
Well G7: changed to 1600 µl

Well G8: changed to 1600 µl
Well G9: changed to 1600 µl
Well G10: changed to 1600 µl
Well G11: changed to 1600 µl
Well G12: changed to 1600 µl
Well H1: changed to 1600 µl
Well H2: changed to 1600 µl
Well H3: changed to 1600 µl
Well H4: changed to 1600 µl
Well H5: changed to 1600 µl
Well H6: changed to 1600 µl
Well H7: changed to 1600 µl
Well H8: changed to 1600 µl
Well H9: changed to 1600 µl
Well H10: changed to 1600 µl
Well H11: changed to 1600 µl
Well H12: changed to 1600 µl

4:54:42 PM 13 Sample Transfer
Source:
Name: DWP 2mL, Labware: dws/plates/dwp96/EP_DWP_2000_1
Destination:
Name: DWP_1mL, Labware: dws/plates/dwp96/EP_DWP_1000_1
Number of samples: 32, replicates: 1
Tool: TM_1000_8, Filter tips: Yes, Liquid: Alcohol 98%
Volume: 750µl, Transfer type: Pipette
Change tips: before each aspiration
Dispose tips into waste.
Options: aspirate 19 mm from well bottom, move -8 mm during aspiration
Changes in liquid types:
Speed aspiration: 1.0mm/sec, Speed dispense: 4.0mm/sec
Liquid offset aspiration: 3.0mm, Liquid offset dispense: -3.0mm
Delay blow: 0ms, Speed blow: 0.4mm/sec
Movement blow: 0%, Initial stroke: 100%
Prewetting: 0 cycles

4:55:02 PM New tip acquired from tip1000f_1, A12
4:55:23 PM Source: DWP 2mL, Position: A1, Well volume: 850.0µl
4:55:23 PM Source: DWP 2mL, Position: B1, Well volume: 850.0µl
4:55:23 PM Source: DWP 2mL, Position: C1, Well volume: 850.0µl
4:55:23 PM Source: DWP 2mL, Position: D1, Well volume: 850.0µl
4:55:23 PM Source: DWP 2mL, Position: E1, Well volume: 850.0µl

Eppendorf AG
Barkhausenweg 1
22339 Hamburg
Germany

4:55:23 PM Source: DWP 2mL, Position: F1, Well volume: 850.0µl
4:55:23 PM Source: DWP 2mL, Position: G1, Well volume: 850.0µl
4:55:23 PM Source: DWP 2mL, Position: H1, Well volume: 850.0µl
4:55:31 PM Destination: DWP_1mL, Position: A1, Well volume: 750.0µl
4:55:31 PM Destination: DWP_1mL, Position: B1, Well volume: 750.0µl
4:55:31 PM Destination: DWP_1mL, Position: C1, Well volume: 750.0µl
4:55:31 PM Destination: DWP_1mL, Position: D1, Well volume: 750.0µl
4:55:31 PM Destination: DWP_1mL, Position: E1, Well volume: 750.0µl
4:55:31 PM Destination: DWP_1mL, Position: F1, Well volume: 750.0µl
4:55:31 PM Destination: DWP_1mL, Position: G1, Well volume: 750.0µl
4:55:31 PM Destination: DWP_1mL, Position: H1, Well volume: 750.0µl
4:55:36 PM Tip disposed.
4:55:53 PM !Dialog Text: Please insert new tips for 1000 µl with filter in slot A2 (tip1000f_1)

WARNING: Please also make sure that you remove all restored tips of the given type.
Reply: 1
4:56:05 PM Location scan: Slot A2, height 99.766 mm
4:56:20 PM Genuine Eppendorf tips found in slot A2
4:56:32 PM Tip Scan Name: tip1000f_1, Labware: dws/tips/tip1000f, Number of tips: 96
4:56:43 PM New tip acquired from tip1000f_1, A1
4:57:04 PM Source: DWP 2mL, Position: A2, Well volume: 850.0µl
4:57:04 PM Source: DWP 2mL, Position: B2, Well volume: 850.0µl
4:57:04 PM Source: DWP 2mL, Position: C2, Well volume: 850.0µl
4:57:04 PM Source: DWP 2mL, Position: D2, Well volume: 850.0µl
4:57:04 PM Source: DWP 2mL, Position: E2, Well volume: 850.0µl
4:57:04 PM Source: DWP 2mL, Position: F2, Well volume: 850.0µl
4:57:04 PM Source: DWP 2mL, Position: G2, Well volume: 850.0µl
4:57:04 PM Source: DWP 2mL, Position: H2, Well volume: 850.0µl
4:57:12 PM Destination: DWP_1mL, Position: A2, Well volume: 750.0µl
4:57:12 PM Destination: DWP_1mL, Position: B2, Well volume: 750.0µl
4:57:12 PM Destination: DWP_1mL, Position: C2, Well volume: 750.0µl
4:57:12 PM Destination: DWP_1mL, Position: D2, Well volume: 750.0µl
4:57:12 PM Destination: DWP_1mL, Position: E2, Well volume: 750.0µl
4:57:12 PM Destination: DWP_1mL, Position: F2, Well volume: 750.0µl
4:57:12 PM Destination: DWP_1mL, Position: G2, Well volume: 750.0µl
4:57:12 PM Destination: DWP_1mL, Position: H2, Well volume: 750.0µl
4:57:17 PM Tip disposed.
4:57:24 PM New tip acquired from tip1000f_1, A2
4:57:46 PM Source: DWP 2mL, Position: A3, Well volume: 850.0µl

4:57:46 PM Source: DWP 2mL, Position: B3, Well volume: 850.0µl
4:57:46 PM Source: DWP 2mL, Position: C3, Well volume: 850.0µl
4:57:46 PM Source: DWP 2mL, Position: D3, Well volume: 850.0µl
4:57:46 PM Source: DWP 2mL, Position: E3, Well volume: 850.0µl
4:57:46 PM Source: DWP 2mL, Position: F3, Well volume: 850.0µl
4:57:46 PM Source: DWP 2mL, Position: G3, Well volume: 850.0µl
4:57:46 PM Source: DWP 2mL, Position: H3, Well volume: 850.0µl
4:57:53 PM Destination: DWP_1mL, Position: A3, Well volume: 750.0µl
4:57:53 PM Destination: DWP_1mL, Position: B3, Well volume: 750.0µl
4:57:53 PM Destination: DWP_1mL, Position: C3, Well volume: 750.0µl
4:57:53 PM Destination: DWP_1mL, Position: D3, Well volume: 750.0µl
4:57:53 PM Destination: DWP_1mL, Position: E3, Well volume: 750.0µl
4:57:53 PM Destination: DWP_1mL, Position: F3, Well volume: 750.0µl
4:57:53 PM Destination: DWP_1mL, Position: G3, Well volume: 750.0µl
4:57:53 PM Destination: DWP_1mL, Position: H3, Well volume: 750.0µl
4:57:59 PM Tip disposed.
4:58:06 PM New tip acquired from tip1000f_1, A3
4:58:27 PM Source: DWP 2mL, Position: A4, Well volume: 850.0µl
4:58:27 PM Source: DWP 2mL, Position: B4, Well volume: 850.0µl
4:58:27 PM Source: DWP 2mL, Position: C4, Well volume: 850.0µl
4:58:27 PM Source: DWP 2mL, Position: D4, Well volume: 850.0µl
4:58:27 PM Source: DWP 2mL, Position: E4, Well volume: 850.0µl
4:58:27 PM Source: DWP 2mL, Position: F4, Well volume: 850.0µl
4:58:27 PM Source: DWP 2mL, Position: G4, Well volume: 850.0µl
4:58:27 PM Source: DWP 2mL, Position: H4, Well volume: 850.0µl
4:58:35 PM Destination: DWP_1mL, Position: A4, Well volume: 750.0µl
4:58:35 PM Destination: DWP_1mL, Position: B4, Well volume: 750.0µl
4:58:35 PM Destination: DWP_1mL, Position: C4, Well volume: 750.0µl
4:58:35 PM Destination: DWP_1mL, Position: D4, Well volume: 750.0µl
4:58:35 PM Destination: DWP_1mL, Position: E4, Well volume: 750.0µl
4:58:35 PM Destination: DWP_1mL, Position: F4, Well volume: 750.0µl
4:58:35 PM Destination: DWP_1mL, Position: G4, Well volume: 750.0µl
4:58:35 PM Destination: DWP_1mL, Position: H4, Well volume: 750.0µl
4:58:40 PM Tip disposed.
Tip usage summary:
tip1000f : 40
tip300f : 40
4:58:59 PM Application ended Application ended successfully

- -

Eppendorf epMotion End of log

Eppendorf AG
Barkhausenweg 1
22339 Hamburg
Germany
