# Supplementary material for: Four-dimensional trapped ion mobility spectrometry lipidomics for high throughput clinical profiling of human blood samples
Source: Nat Commun. 2023 Feb 20;14:937. doi: 10.1038/s41467-023-36520-1 (PMC9941096; doi:10.1038/s41467-023-36520-1)
Supplement: Supplementary file 15 — Supplementary Data 12 [file 41467_2023_36520_MOESM15_ESM.pdf]

Eppendorf AG
Barkhausenweg 1
22339 Hamburg
Germany

9/13/2022

**User**	user
**Device**	5073JK706157
**Logfile**	32_sample_solving_r1_2022_08_26_18_15_45_5073JK706157
**Logfile Path**	Home
**Logfile Created**	
**Software version**	Device: 18.4 Server: 40.7.2.8
**Application**	
**Created**	
**Last modified**	
**Path**	Home
**Comment**	

Eppendorf epMotion

- -

Method name and path :

Date of Run:
Last edit :
Firmware version : epMotion 18.04
Software version : 40.7.2.8
TIME----- COMMAND------------ INFORMATION-------------------------------
6:15:35 PM Program Init
6:15:35 PM Program Collect
6:15:41 PM !Dialog Text: Number of samples
Reply: 1
Answer: 32
6:15:45 PM Program Check
6:15:45 PM Application runner settings
Levels: ON
Tips: ON
Locations: OFF

Eppendorf AG
Barkhausenweg 1
22339 Hamburg
Germany

Vessel caps: OFF
Auto tool selection: OFF
6:15:57 PM HEPA unit: OFF
6:15:57 PM !!! WARNING !!! Device setup is invalid. Please contact service.
6:15:57 PM Level Name: DWP 1ml, Labware: dws/plates/dwp96/EP_DWP_1000_1
Position: A1, Volume: 0µl
Position: A2, Volume: 0µl
Position: A3, Volume: 0µl
Position: A4, Volume: 0µl
Position: B1, Volume: 0µl
Position: B2, Volume: 0µl
Position: B3, Volume: 0µl
Position: B4, Volume: 0µl
Position: C1, Volume: 0µl
Position: C2, Volume: 0µl
Position: C3, Volume: 0µl
Position: C4, Volume: 0µl
Position: D1, Volume: 0µl
Position: D2, Volume: 0µl
Position: D3, Volume: 0µl
Position: D4, Volume: 0µl
Position: E1, Volume: 0µl
Position: E2, Volume: 0µl
Position: E3, Volume: 0µl
Position: E4, Volume: 0µl
Position: F1, Volume: 0µl
Position: F2, Volume: 0µl
Position: F3, Volume: 0µl
Position: F4, Volume: 0µl
Position: G1, Volume: 0µl
Position: G2, Volume: 0µl
Position: G3, Volume: 0µl
Position: G4, Volume: 0µl
Position: H1, Volume: 0µl
Position: H2, Volume: 0µl
Position: H3, Volume: 0µl
Position: H4, Volume: 0µl
6:16:01 PM Location scan: Slot A2, height 99.843 mm
6:16:15 PM Genuine Eppendorf tips found in slot A2
6:16:28 PM Tip Scan Name: tip1000f_1, Labware: dws/tips/tip1000f, Number of tips: 40

Eppendorf AG
Barkhausenweg 1
22339 Hamburg
Germany

6:16:32 PM Location scan: Slot B1, height 59.048 mm
6:16:47 PM Genuine Eppendorf tips found in slot B1
6:17:00 PM Tip Scan Name: tip50f_1, Labware: dws/tips/tip50f, Number of tips: 56
6:17:03 PM Location scan: Slot B2, height 58.915 mm
6:17:18 PM Genuine Eppendorf tips found in slot B2
6:17:31 PM Tip Scan Name: tip300f_1, Labware: dws/tips/tip300f, Number of tips: 32
6:17:42 PM Level Name: solvents 2, Labware: dws/th/solvents
Position: 4, Volume: 74223.7µl
Position: 5, Volume: 24577.9µl
6:17:42 PM Level Name: Injektion plate, Labware: dws/plates/pcr96/NGS_EP_TT_PCR_150
Position: A1, Volume: 0µl
Position: A2, Volume: 0µl
Position: A3, Volume: 0µl
Position: A4, Volume: 0µl
Position: B1, Volume: 0µl
Position: B2, Volume: 0µl
Position: B3, Volume: 0µl
Position: B4, Volume: 0µl
Position: C1, Volume: 0µl
Position: C2, Volume: 0µl
Position: C3, Volume: 0µl
Position: C4, Volume: 0µl
Position: D1, Volume: 0µl
Position: D2, Volume: 0µl
Position: D3, Volume: 0µl
Position: D4, Volume: 0µl
Position: E1, Volume: 0µl
Position: E2, Volume: 0µl
Position: E3, Volume: 0µl
Position: E4, Volume: 0µl
Position: F1, Volume: 0µl
Position: F2, Volume: 0µl
Position: F3, Volume: 0µl
Position: F4, Volume: 0µl
Position: G1, Volume: 0µl
Position: G2, Volume: 0µl
Position: G3, Volume: 0µl
Position: G4, Volume: 0µl
Position: H1, Volume: 0µl
Position: H2, Volume: 0µl

Position: H3, Volume: 0µl
Position: H4, Volume: 0µl
6:17:46 PM Tool Scan Position: Gripper, Code: N/A
6:17:49 PM Tool Scan Position: T1, Code: D_50_8, serial number: 1002123
6:17:52 PM Tool Scan Position: T2, Code: D_300_8, serial number: 224322K
6:17:55 PM Tool Scan Position: T3, Code: D_1000_8, serial number: 421352I
6:18:00 PM 1 User Intervention Comment: place 1ml plate on TMX position
place Injection plate on C1 position
Alarm: On
6:18:08 PM continue
6:18:08 PM 2 Comment solve in 360µl MeOH
6:18:08 PM 3 Reagent Transfer
Source:
Name: solvents 2, Labware: dws/th/solvents
Destination:
Name: DWP 1ml, Labware: dws/plates/dwp96/EP_DWP_1000_1
Number of samples: 32
Tool: TM_1000_8, Filter tips: Yes, Liquid: Alcohol 98%
Volume: 360µl, Transfer type: Pipette
Change tips: when command finished after 2 aspirations
Dispose tips into waste.
6:18:21 PM New tip acquired from tip1000f_1, A8
6:18:35 PM Source: solvents 2, Position: 4, Well volume: 71343.7µl
6:18:41 PM Destination: DWP 1ml, Position: A1, Well volume: 360.0µl
6:18:41 PM Destination: DWP 1ml, Position: B1, Well volume: 360.0µl
6:18:41 PM Destination: DWP 1ml, Position: C1, Well volume: 360.0µl
6:18:41 PM Destination: DWP 1ml, Position: D1, Well volume: 360.0µl
6:18:41 PM Destination: DWP 1ml, Position: E1, Well volume: 360.0µl
6:18:41 PM Destination: DWP 1ml, Position: F1, Well volume: 360.0µl
6:18:41 PM Destination: DWP 1ml, Position: G1, Well volume: 360.0µl
6:18:41 PM Destination: DWP 1ml, Position: H1, Well volume: 360.0µl
6:18:48 PM Source: solvents 2, Position: 4, Well volume: 68463.7µl
6:18:54 PM Destination: DWP 1ml, Position: A2, Well volume: 360.0µl
6:18:54 PM Destination: DWP 1ml, Position: B2, Well volume: 360.0µl
6:18:54 PM Destination: DWP 1ml, Position: C2, Well volume: 360.0µl
6:18:54 PM Destination: DWP 1ml, Position: D2, Well volume: 360.0µl
6:18:54 PM Destination: DWP 1ml, Position: E2, Well volume: 360.0µl
6:18:54 PM Destination: DWP 1ml, Position: F2, Well volume: 360.0µl
6:18:54 PM Destination: DWP 1ml, Position: G2, Well volume: 360.0µl
6:18:54 PM Destination: DWP 1ml, Position: H2, Well volume: 360.0µl

Eppendorf AG
Barkhausenweg 1
22339 Hamburg
Germany

6:19:01 PM Tip disposed.
6:19:08 PM New tip acquired from tip1000f_1, A9
6:19:23 PM Source: solvents 2, Position: 4, Well volume: 65583.7µl
6:19:29 PM Destination: DWP 1ml, Position: A3, Well volume: 360.0µl
6:19:29 PM Destination: DWP 1ml, Position: B3, Well volume: 360.0µl
6:19:29 PM Destination: DWP 1ml, Position: C3, Well volume: 360.0µl
6:19:29 PM Destination: DWP 1ml, Position: D3, Well volume: 360.0µl
6:19:29 PM Destination: DWP 1ml, Position: E3, Well volume: 360.0µl
6:19:29 PM Destination: DWP 1ml, Position: F3, Well volume: 360.0µl
6:19:29 PM Destination: DWP 1ml, Position: G3, Well volume: 360.0µl
6:19:29 PM Destination: DWP 1ml, Position: H3, Well volume: 360.0µl
6:19:35 PM Source: solvents 2, Position: 4, Well volume: 62703.7µl
6:19:42 PM Destination: DWP 1ml, Position: A4, Well volume: 360.0µl
6:19:42 PM Destination: DWP 1ml, Position: B4, Well volume: 360.0µl
6:19:42 PM Destination: DWP 1ml, Position: C4, Well volume: 360.0µl
6:19:42 PM Destination: DWP 1ml, Position: D4, Well volume: 360.0µl
6:19:42 PM Destination: DWP 1ml, Position: E4, Well volume: 360.0µl
6:19:42 PM Destination: DWP 1ml, Position: F4, Well volume: 360.0µl
6:19:42 PM Destination: DWP 1ml, Position: G4, Well volume: 360.0µl
6:19:42 PM Destination: DWP 1ml, Position: H4, Well volume: 360.0µl
6:19:49 PM Tip disposed.
6:19:53 PM 4 User Intervention Comment: place silikone matt on 1ml DWP
Alarm: On
6:20:34 PM continue
6:20:34 PM 5 Comment vortex for 5min
6:20:35 PM 6 Thermomixer Speed: 1200rpm, Time: 05:00
Temperature: 20°C
Current Temperature: 21°C
6:20:35 PM 7 User Intervention Comment: take off silikone matt of 1ml plate
Alarm: On
6:25:48 PM continue
6:25:48 PM 8 Comment add 8 µl H2O
6:25:48 PM 9 Reagent Transfer
Source:
Name: solvents 2, Labware: dws/th/solvents
Destination:
Name: Injektion plate, Labware: dws/plates/pcr96/NGS_EP_TT_PCR_150
Number of samples: 32
Tool: TM_50_8, Filter tips: Yes, Liquid: Water
Volume: 8.0µl, Transfer type: Pipette

Eppendorf AG
Barkhausenweg 1
22339 Hamburg
Germany

Change tips: when command finished after 6 aspirations
Dispose tips into waste.
Options: dispense 5 from liquid level, follow liquid level change
6:26:07 PM New tip acquired from tip50f_1, A6
6:26:11 PM Source: solvents 2, Position: 5, Well volume: 24513.9µl
6:26:15 PM Destination: Injektion plate, Position: A1, Well volume: 8.0µl
6:26:15 PM Destination: Injektion plate, Position: B1, Well volume: 8.0µl
6:26:15 PM Destination: Injektion plate, Position: C1, Well volume: 8.0µl
6:26:15 PM Destination: Injektion plate, Position: D1, Well volume: 8.0µl
6:26:15 PM Destination: Injektion plate, Position: E1, Well volume: 8.0µl
6:26:15 PM Destination: Injektion plate, Position: F1, Well volume: 8.0µl
6:26:15 PM Destination: Injektion plate, Position: G1, Well volume: 8.0µl
6:26:15 PM Destination: Injektion plate, Position: H1, Well volume: 8.0µl
6:26:19 PM Source: solvents 2, Position: 5, Well volume: 24449.9µl
6:26:22 PM Destination: Injektion plate, Position: A2, Well volume: 8.0µl
6:26:22 PM Destination: Injektion plate, Position: B2, Well volume: 8.0µl
6:26:22 PM Destination: Injektion plate, Position: C2, Well volume: 8.0µl
6:26:22 PM Destination: Injektion plate, Position: D2, Well volume: 8.0µl
6:26:22 PM Destination: Injektion plate, Position: E2, Well volume: 8.0µl
6:26:22 PM Destination: Injektion plate, Position: F2, Well volume: 8.0µl
6:26:22 PM Destination: Injektion plate, Position: G2, Well volume: 8.0µl
6:26:22 PM Destination: Injektion plate, Position: H2, Well volume: 8.0µl
6:26:26 PM Source: solvents 2, Position: 5, Well volume: 24385.9µl
6:26:29 PM Destination: Injektion plate, Position: A3, Well volume: 8.0µl
6:26:29 PM Destination: Injektion plate, Position: B3, Well volume: 8.0µl
6:26:29 PM Destination: Injektion plate, Position: C3, Well volume: 8.0µl
6:26:29 PM Destination: Injektion plate, Position: D3, Well volume: 8.0µl
6:26:29 PM Destination: Injektion plate, Position: E3, Well volume: 8.0µl
6:26:29 PM Destination: Injektion plate, Position: F3, Well volume: 8.0µl
6:26:29 PM Destination: Injektion plate, Position: G3, Well volume: 8.0µl
6:26:29 PM Destination: Injektion plate, Position: H3, Well volume: 8.0µl
6:26:33 PM Source: solvents 2, Position: 5, Well volume: 24321.9µl
6:26:36 PM Destination: Injektion plate, Position: A4, Well volume: 8.0µl
6:26:36 PM Destination: Injektion plate, Position: B4, Well volume: 8.0µl
6:26:36 PM Destination: Injektion plate, Position: C4, Well volume: 8.0µl
6:26:36 PM Destination: Injektion plate, Position: D4, Well volume: 8.0µl
6:26:36 PM Destination: Injektion plate, Position: E4, Well volume: 8.0µl
6:26:36 PM Destination: Injektion plate, Position: F4, Well volume: 8.0µl
6:26:36 PM Destination: Injektion plate, Position: G4, Well volume: 8.0µl
6:26:36 PM Destination: Injektion plate, Position: H4, Well volume: 8.0µl

6:26:42 PM Tip disposed.
6:26:46 PM 10 Comment for sample injection: 72µl lipid extract
6:26:46 PM 11 Sample Transfer
Source:
Name: DWP 1ml, Labware: dws/plates/dwp96/EP_DWP_1000_1
Destination:
Name: Injektion plate, Labware: dws/plates/pcr96/NGS_EP_TT_PCR_150
Number of samples: 32, replicates: 1
Tool: TM_300_8, Filter tips: Yes, Liquid: Alcohol 98%
Volume: 72.0µl, Transfer type: Pipette
Change tips: before each aspiration
Dispose tips into waste.
6:27:08 PM New tip acquired from tip300f_1, A9
6:27:19 PM Source: DWP 1ml, Position: A1, Well volume: 288.0µl
6:27:19 PM Source: DWP 1ml, Position: B1, Well volume: 288.0µl
6:27:19 PM Source: DWP 1ml, Position: C1, Well volume: 288.0µl
6:27:19 PM Source: DWP 1ml, Position: D1, Well volume: 288.0µl
6:27:19 PM Source: DWP 1ml, Position: E1, Well volume: 288.0µl
6:27:19 PM Source: DWP 1ml, Position: F1, Well volume: 288.0µl
6:27:19 PM Source: DWP 1ml, Position: G1, Well volume: 288.0µl
6:27:19 PM Source: DWP 1ml, Position: H1, Well volume: 288.0µl
6:27:23 PM Destination: Injektion plate, Position: A1, Well volume: 80.0µl
6:27:23 PM Destination: Injektion plate, Position: B1, Well volume: 80.0µl
6:27:23 PM Destination: Injektion plate, Position: C1, Well volume: 80.0µl
6:27:23 PM Destination: Injektion plate, Position: D1, Well volume: 80.0µl
6:27:23 PM Destination: Injektion plate, Position: E1, Well volume: 80.0µl
6:27:23 PM Destination: Injektion plate, Position: F1, Well volume: 80.0µl
6:27:23 PM Destination: Injektion plate, Position: G1, Well volume: 80.0µl
6:27:23 PM Destination: Injektion plate, Position: H1, Well volume: 80.0µl
6:27:30 PM Tip disposed.
6:27:37 PM New tip acquired from tip300f_1, A10
6:27:48 PM Source: DWP 1ml, Position: A2, Well volume: 288.0µl
6:27:48 PM Source: DWP 1ml, Position: B2, Well volume: 288.0µl
6:27:48 PM Source: DWP 1ml, Position: C2, Well volume: 288.0µl
6:27:48 PM Source: DWP 1ml, Position: D2, Well volume: 288.0µl
6:27:48 PM Source: DWP 1ml, Position: E2, Well volume: 288.0µl
6:27:48 PM Source: DWP 1ml, Position: F2, Well volume: 288.0µl
6:27:48 PM Source: DWP 1ml, Position: G2, Well volume: 288.0µl
6:27:48 PM Source: DWP 1ml, Position: H2, Well volume: 288.0µl
6:27:52 PM Destination: Injektion plate, Position: A2, Well volume: 80.0µl

6:27:52 PM Destination: Injektion plate, Position: B2, Well volume: 80.0µl
6:27:52 PM Destination: Injektion plate, Position: C2, Well volume: 80.0µl
6:27:52 PM Destination: Injektion plate, Position: D2, Well volume: 80.0µl
6:27:52 PM Destination: Injektion plate, Position: E2, Well volume: 80.0µl
6:27:52 PM Destination: Injektion plate, Position: F2, Well volume: 80.0µl
6:27:52 PM Destination: Injektion plate, Position: G2, Well volume: 80.0µl
6:27:52 PM Destination: Injektion plate, Position: H2, Well volume: 80.0µl
6:27:59 PM Tip disposed.
6:28:07 PM New tip acquired from tip300f_1, A11
6:28:17 PM Source: DWP 1ml, Position: A3, Well volume: 288.0µl
6:28:17 PM Source: DWP 1ml, Position: B3, Well volume: 288.0µl
6:28:17 PM Source: DWP 1ml, Position: C3, Well volume: 288.0µl
6:28:17 PM Source: DWP 1ml, Position: D3, Well volume: 288.0µl
6:28:17 PM Source: DWP 1ml, Position: E3, Well volume: 288.0µl
6:28:17 PM Source: DWP 1ml, Position: F3, Well volume: 288.0µl
6:28:17 PM Source: DWP 1ml, Position: G3, Well volume: 288.0µl
6:28:17 PM Source: DWP 1ml, Position: H3, Well volume: 288.0µl
6:28:21 PM Destination: Injektion plate, Position: A3, Well volume: 80.0µl
6:28:21 PM Destination: Injektion plate, Position: B3, Well volume: 80.0µl
6:28:21 PM Destination: Injektion plate, Position: C3, Well volume: 80.0µl
6:28:21 PM Destination: Injektion plate, Position: D3, Well volume: 80.0µl
6:28:21 PM Destination: Injektion plate, Position: E3, Well volume: 80.0µl
6:28:21 PM Destination: Injektion plate, Position: F3, Well volume: 80.0µl
6:28:21 PM Destination: Injektion plate, Position: G3, Well volume: 80.0µl
6:28:21 PM Destination: Injektion plate, Position: H3, Well volume: 80.0µl
6:28:28 PM Tip disposed.
6:28:36 PM New tip acquired from tip300f_1, A12
6:28:46 PM Source: DWP 1ml, Position: A4, Well volume: 288.0µl
6:28:47 PM Source: DWP 1ml, Position: B4, Well volume: 288.0µl
6:28:47 PM Source: DWP 1ml, Position: C4, Well volume: 288.0µl
6:28:47 PM Source: DWP 1ml, Position: D4, Well volume: 288.0µl
6:28:47 PM Source: DWP 1ml, Position: E4, Well volume: 288.0µl
6:28:47 PM Source: DWP 1ml, Position: F4, Well volume: 288.0µl
6:28:47 PM Source: DWP 1ml, Position: G4, Well volume: 288.0µl
6:28:47 PM Source: DWP 1ml, Position: H4, Well volume: 288.0µl
6:28:51 PM Destination: Injektion plate, Position: A4, Well volume: 80.0µl
6:28:51 PM Destination: Injektion plate, Position: B4, Well volume: 80.0µl
6:28:51 PM Destination: Injektion plate, Position: C4, Well volume: 80.0µl
6:28:51 PM Destination: Injektion plate, Position: D4, Well volume: 80.0µl
6:28:51 PM Destination: Injektion plate, Position: E4, Well volume: 80.0µl

Eppendorf AG
Barkhausenweg 1
22339 Hamburg
Germany

6:28:51 PM Destination: Injektion plate, Position: F4, Well volume: 80.0µl
6:28:51 PM Destination: Injektion plate, Position: G4, Well volume: 80.0µl
6:28:51 PM Destination: Injektion plate, Position: H4, Well volume: 80.0µl
6:28:58 PM Tip disposed.
Tip usage summary:
tip1000f : 16
tip300f : 32
tip50f : 8
6:29:15 PM Application ended Application ended successfully
- -
Eppendorf epMotion End of log
