# Supplementary material for: Four-dimensional trapped ion mobility spectrometry lipidomics for high throughput clinical profiling of human blood samples
Source: Nat Commun. 2023 Feb 20;14:937. doi: 10.1038/s41467-023-36520-1 (PMC9941096; doi:10.1038/s41467-023-36520-1)
Supplement: Supplementary file 18 — Supplementary Data 15 [file 41467_2023_36520_MOESM18_ESM.pdf]

```
import numpy as np
import pandas as pd


def find_matches_by_threshold(array_1, array_2, mz_tol=0.002, ccs_tol=0.2,
 rt_tol=0.2):
    """
    Given two numpy arrays find matches between the elements of the two
     such that the M/Z, RT,
    and CCS values of the two elements are within a given tolerance level.
        :param array_1: first array with M/Z, RT and CCS values
        :param array_2: second array with M/Z, RT and CCS values
        :param mz_tol:  tolerance accepted for M/Z values
        :param rt_tol:  tolerance accepted for RT values
        :param ccs_tol: tolerance accepted for CCS values
        :return: lists containing the indexes of the overlapping indexes in
         the respectively other array
        """

    # Initiate empty list which will store the matches found for each
     element in array_1 with the elements in array_2
    overlap = []

    for i in range(len(array_1)):
        # Find index for elements where MZ values of elements in the two
         arrays are within the absolute tolerance range
        mz = np.isclose(array_1[i, 0], array_2[:, 0], atol=mz_tol)
        # Find index for elements where RT values of elements in the two
         arrays are within the absolute tolerance range
        rt = np.isclose(array_1[i, 1], array_2[:, 1], atol=rt_tol)
        # Find index for elements where CCS values of elements in the two
         arrays are within the absolute tolerance range
        ccs = np.isclose(array_1[i, 2], array_2[:, 2], atol=ccs_tol)
        # Find indexes which are in all three of the above arrays
        matches = np.where(np.logical_and(mz, np.logical_and(rt,
         ccs)))[0].tolist()
        # If an element matches with one or multiple elements in the other
         array concatenate them into a single string
        if matches:
            matches = ', '.join([str(ind) for ind in matches])
        # If there is no match found return '-'
        else:
            matches = '-'
        # Append the matches to the overlap list
        overlap.append(matches)
    return overlap


if __name__ == "__main__":
    # ----------------------------- #
    # READ DATA:                    #
    # ----------------------------- #

    # Sample array 1
    # Path to file containing the dilution data
```

```
dilution_file = "raw_data/210813 repetition vs dilution raw data -
 SUF.xlsx"
# Read data to Dataframe
dilution = pd.read_excel(dilution_file, 0)
# Select relevant columns and cast them to numpy array where each
 element in the array contains three values:
#   [[MZ, RT, CCS], ...]
dilution_val = dilution[["m/z meas.", "RT [min]", "CCS (Å²)"]].values

# Sample array 2
# Path to file containing the repetition experiment data
repetition_file = "raw_data/210813 repetition - MeOH - final
 annotation.xlsx"
# Read data to Dataframe
repetition = pd.read_excel(repetition_file, 0)
# Select relevant columns and cast them to numpy array where each
 element in the array contains three values:
#   [[MZ, RT, CCS], ...]
repetition_val = repetition[["m/z meas.", "RT [min]", "CCS
 (Å²)"]].values

# ----------------------------- #
# START PROCESSING:             #
# ----------------------------- #
# Define tolerance with which MZ, RT and CCS of two elements
mz_tolerance = 0.002
rt_tolerance = 0.1
ccs_tolerance = 0.2

# Find matches of dilution data in repetition data
matches_dilution = find_matches_by_threshold(dilution_val,
 repetition_val,
                                             mz_tol=mz_tolerance,
                                              ccs_tol=ccs_tolerance,
                                              rt_tol=rt_tolerance)
dilution[f"Dilution (mz_tol={mz_tolerance}, ccs_tol={ccs_tolerance},
 rt_tol={rt_tolerance})"] = matches_dilution

# Find matches of repetition data in dilution data
matches_repetition = find_matches_by_threshold(repetition_val,
 dilution_val,
                                               mz_tol=mz_tolerance,
                                               ccs_tol=ccs_tolerance,
                                               rt_tol=rt_tolerance)
repetition[f"Repetition (mz_tol={mz_tolerance},
 ccs_tol={ccs_tolerance}, "
           f"rt_tol={rt_tolerance})"] = matches_repetition

# ----------------------------- #
# WRITE RESULTS TO EXCEL FILE:  #
# ----------------------------- #
# Specify a path under which you want to save your processed data
results_file = "processed_data/210813 repetition - MeOH - final
 annotation_processed_test.xlsx"
# Save the results
```

```
with pd.ExcelWriter(results_file) as writer:
    dilution.to_excel(writer, sheet_name='dilution')
    repetition.to_excel(writer, sheet_name='210813 minus
     MeOH—annotated')
```
